# Incidence of Induced Abortion in Uganda, 2013: New Estimates Since 2003

**DOI:** 10.1371/journal.pone.0165812

**Published:** 2016-11-01

**Authors:** Elena Prada, Lynn M. Atuyambe, Nakeisha M. Blades, Justine N. Bukenya, Christopher Garimoi Orach, Akinrinola Bankole

**Affiliations:** 1 Independent Consultant, Bogotá, Colombia, South America; 2 Makerere University, School of Public Health, Kampala, Uganda; 3 Guttmacher Institute, 125 Maiden Lane, New York, New York, United States of America; NHS lothian and University of Edinburgh, UNITED KINGDOM

## Abstract

**Background:**

In Uganda, abortion is permitted only when the life of a woman is in danger. This restriction compels the perpetuation of the practice in secrecy and often under unsafe conditions. In 2003, 294,000 induced abortions were estimated to occur each year in Uganda. Since then, no other research on abortion incidence has been conducted in the country.

**Methods:**

Data from 418 health facilities were used to estimate the number and rate of induced abortion in 2013. An indirect estimation methodology was used to calculate the annual incidence of induced abortions ─ nationally and by major regions. The use of a comparable methodology in an earlier study permits assessment of trends between 2003 and 2013.

**Results:**

In 2013, an estimated 128,682 women were treated for abortion complications and an estimated 314,304 induced abortions occurred, both slightly up from 110,000 and 294,000 in 2003, respectively. The national abortion rate was 39 abortions per 1,000 women aged 15–49, down from 51 in 2003. Regional variation in abortion rates is very large, from as high as an estimated 77 per 1,000 women 15–49 in Kampala region, to as low as 18 per 1,000 women in Western region. The overall pregnancy rate also declined from 326 to 288; however the proportion of pregnancies that were unintended increased slightly, from 49% to 52%.

**Conclusion:**

Unsafe abortion remains a major problem confronting Ugandan women. Although the overall pregnancy rate and the abortion rate declined in the past decade, the majority of pregnancies to Ugandan women are still unintended. These findings reflect the increase in the use of modern contraception but also suggest that a large proportion of women are still having difficulty practicing contraception effectively. Improved access to contraceptive services and abortion-related care are still needed.

## Introduction

The Ugandan Penal Code of 1950 indicates criminal penalties for anyone who obtains an abortion or contributes to the procurement of one [1]. There is one exception permitted–if the abortion procedure is to save the woman’s life [2]. Court cases have since expanded the interpretation of the life indication to include preservation of a woman’s physical and mental health as well [3]. The Ministry of Health’s (MOH) National Policy Guidelines and Service Standards for Sexual and Reproductive Health and Rights specify a number of health grounds under which abortion is permitted, including rape, incest and defilement, or if the woman has HIV; these policies also expand abortion provision allowing access to the service at facilities ranging from Health Center IVs through the referral hospital level [1,4]. However, it is difficult to ascertain whether an apparent clinical life risk will be seen as such under the law, especially if there are conflicting medical opinions on the level of risk a woman is facing. Many providers and women are also still unaware of the expansions on the abortion law [4]. This lack of clarity has made legal abortion difficult to obtain and to provide, and has allowed unsafe abortion to continue, with serious consequences for women and society. In 2003 it was estimated that about 294,000 abortions, most of which were unsafe, took place in Uganda [5].

Unsafe abortion is a known contributor to maternal mortality in Uganda, although its impact seems to have decreased over time. In 2006, the MOH estimated that abortion-related causes accounted for 26% of maternal deaths [6]. A 2007 study, conducted in 553 health facilities to monitor provision of obstetric care services, found that complications from abortion were directly responsible for 11% of maternal deaths [7]. A more recent estimate, reported by the MOH in the 2010–2015 Strategic Plan for the Health Sector, suggests that 8% of maternal deaths were due to unsafe abortion [8].

Unsafe abortion also largely impacts abortion morbidity. Unsafe procedures, including oral or intravaginal introduction of herbs, caustic substances, drugs, and/or sharp objects, result in complications that can be quite severe and even result in permanent damage to the body [9–12]. In 2003, it was estimated that over 85,000 women were treated for complications arising from abortions [5]; the aforementioned 2007 health facility study also found that about 40% of admissions for emergency obstetric care were the result of unsafe abortions [7]. Treatment of complications from unsafe abortion consumes a significant portion of the total expenditure for reproductive health in Uganda. In 2010, an estimated $13.9 million (in US dollars) was spent towards the provision of post abortion care (PAC) services. This corresponds to 4.1% of the total government expenditure on health, which is estimated at $350 million (in US dollars) [13].

Measures of fertility preferences, contraceptive use, limited contraceptive availability, and unmet need for contraception suggest that the level of abortion in Uganda is likely to remain high [14]. On average, Ugandan women have two more children than they desire. Further, 43% of all births in the five years preceding the 2011 Uganda Demographic and Health Survey (UDHS) were reported as unplanned [15]. Use of modern contraceptive methods (as categorized by the UDHS) among Ugandan married women has increased in the past decade from 14% to 26%, but the level is still low compared to other developing countries in Sub-Saharan Africa, where as much as 33% to 64% of married women report use of modern methods [16]. Unmet need for contraception—that is, the percent of women not using any method of contraception among those who either do not want a child soon or do not want any (more) children—has remained high during the past decade: 38% of married women and 45% of unmarried women had an unmet need for contraception in 2011 [15].

In this article, we present new national and regional estimates of induced abortion incidence for 2013, using the same indirect method employed in the 2003 study: the Abortion Incidence Complications Method (AICM). Using this method allows us to assess trends in induced abortion and of unintended pregnancy and its outcomes (unplanned births, abortion and miscarriage) in Uganda over the past decade. We also examine patterns of hospitalization due to abortion-related complications, a key indicator of morbidity resulting from unsafe abortion.

## Materials and Methods

The study design and protocols used in this study were reviewed and approved by the Higher Degrees, Research and Ethics Committee, Makerere University, School of Public Health; by the Ugandan National Council of Science and Technology, and by the Guttmacher Institute’s Institutional Review Board. Informed written consent was obtained from all participants. No minors were involved in this study. All respondents were interviewed in their professional capacities and were not asked to provide any personally identifying information about themselves or patients.

### Data Sources

The AICM relies on two main data sources: A survey of health facilities and a survey of health professionals knowledgeable about the conditions of abortion service provision in the country [17]. The first survey provides information on the number of women treated in the country’s health facilities for abortion complications in a given period. The second survey provides information needed to estimate the proportion of women obtaining abortions who have complications and who obtain treatment at a medical facility.

Questionnaires for both surveys were pretested in June 2013; the final surveys were fielded from July to August 2013 by a team of 33 interviewers and 12 supervisors (who also conducted interviews with health professionals). The sample designs for these two surveys are described below. Other data sources used include the 2000/01 and 2011 UDHS [15,18], and the 2015 United Nations population data [19]. These sources provided data on contraceptive use, planning status of births, unmet need for contraception, fertility rates and the female population by age groups. The 2000/01 and 2011 UDHS were large-scale surveys with nationally representative samples of women aged 15–49 (7,246 in 2000/01 and 8,674 in 2011).

### Sample Design of the Health Facilities Survey (HFS)

All public, private and non-governmental organization (NGO) health facilities considered likely to provide post-abortion medical care for spontaneous and induced abortion complication patients were included in the sample frame. Health Centers (HC) at levels I and II were excluded because they provide only primary health care and refer abortion complication cases to higher-level facilities. Facilities affiliated with the armed forces or prisons, and facilities that provided specialized treatment such as psychiatric and ophthalmological services were also excluded as they were unlikely to provide PAC services.

A list of 1,833 health facilities was obtained from the MOH. The universe of facilities was later adjusted to include an additional 462 private midwives operations registered with the Private Midwives Association and National Midwives and Nurses’ Council that were not included in the original MOH list, but that were identified after fielding.

As hospitals represent primary providers of PAC services, all hospitals in the country were included in the sample. For the other types of facilities–HC IVs, HC IIIs and private midwives–we used a two-step stratified sample design. We first selected districts within each of the ten regions and then selected a proportion of these types of facilities in the selected districts. Selection of districts per region was based on population projections for 2013. Districts within a region were first listed according to population size in ascending order, and then grouped into four strata based on the cumulative distribution of the population size. We randomly selected one district per strata, yielding 36 districts ─four districts in each of the nine regions plus Kampala, which is itself a region. Of all the known facilities in these 37 sampled districts, 60% of HC IV, 30% of HC III and 50% of private midwives were selected into the sample.

Four-hundred seventy-three facilities were selected into the sample. Thirty-four health units were later excluded from this list: one was determined a duplicate and 33 were either closed, did not provide PAC, or were impossible to locate. Of the remaining 439, 48% were government-owned and 52% were private facilities, NGOs, or facilities run by private midwives. Thirty-one percent were hospitals, 11% were HC IVs, 39% were HC III and 18% were units operated by private midwives. Of the selected sample, 418 facilities were interviewed, resulting in an overall response rate of 95%. The response rate varied by type of facility: from 91% of hospitals to 100% in HC IVs (Table 1). There were only three regions with a response rate less than 100%: East Central (94%), Kampala (79%), South West (98%) and Western (96%) (not shown). Reasons for refusal included not having time to participate, religious grounds, requests for remuneration (respondents were not compensated for participation), and concerns about participating in a study on this topic.

**Table 1:** Distribution of health facilities: universe and sample selected for the Health Facility Survey, Uganda 2013.

	All Health Facilities			Sample			Health Facilities Interviewed			Response Rate		
Type of facility	Gov.	NGO/ Private	Total	Gov.	NGO/ Private	Total	Gov.	NGO/ Private	Total	Gov.	NGO/ Private	Total
Total*	**1,094**	**1,180**	**2,274**	**212**	**227**	**439**	**210**	**208**	**418**	**99.1**	**91.6**	**95.2**
Hospital	60	73	133	58	79	137	57	68	125	98.3	86.1	91.2
Health Center IV	164	23	187	42	7	49	42	7	49	100.0	100.0	100.0
Health Center III	870	373	1,243	112	61	173	111	58	169	99.1	95.1	97.7
Private Midwives†	‘-	711	711	‘-	80	80	‘-	75	75	‘-	93.8	93.8

Abbreviations: Gov = government; NGO = Non-Governmental Organizations

* A total of 34 health facilities were excluded from the initial list: one duplicate, while 33 were either closed, did not provide PAC, or were impossible to locate.

† Number registered with the Uganda Private Midwives Associations (UPMA).

Surveys were answered by the senior staff member most knowledgeable about the facility’s provision of PAC services. These respondents included medical doctors, medical officers, nurses, and trained midwives involved in the provision of PAC services. These professionals had an average of 14 years of experience in their current profession and six years of experience working at their current facility (not shown).

### Health Professionals Survey

The Health Professionals Survey (HPS) was conducted among experts in the field of reproductive health in Uganda to obtain their opinions about abortion provision and PAC services. A list of professionals likely to be familiar with the topic was prepared by the in-country team in consultation with experts from professional organizations and international organizations in the field of sexual and reproductive health. This list included health care workers not interviewed in the HFS, policy makers, advocates, researchers and NGO staff. All were selected based on their affiliation, experience and expertise.

The questionnaire was similar to the 2003 study instrument, but also included some questions to explore respondents’ views regarding the availability and use of misoprostol for induced abortion in Uganda. This topic was not examined in the earlier study as the medication was not known to be used at that time in the country. A total of 147 health professionals were selected and interviewed. Respondents represented 15 districts across the country. All HPS respondents had some knowledge of or experience in rural areas, and reported an average of 11 years work experience in the countryside. Forty-three percent of HPS respondents were currently working exclusively in rural or in both rural and urban areas, while an additional 20% had worked in a rural environment within the past five years (not shown).

### Methodology for Estimating Abortion Incidence

#### Weighting the HFS data

The HFS data were weighted to project the results nationally. The weights factored in the probability of selection and of non-response according to region and facility type. Facilities that did not provide PAC were also removed from the facility universe. The weighting factor for a given facility type was the inverse of the sampling proportion multiplied by the proportion of completed interviews among sampled facilities of that type.

#### Calculating the number of women treated for abortion complications

HFS respondents were asked whether their facility provided treatment of spontaneous or induced abortion complications; if they responded affirmatively, they were asked to report the following: 1) the number of women who received PAC as outpatients in a typical month; 2) the number of women who received PAC as outpatients in the past month; 3) the number of women who received PAC as inpatients in a typical month; and 4) the number of women who received PAC as inpatients in the past month (S1 Appendix).

Respondents were asked about a typical or average month in addition to the past month to differentiate from seasonal peaks in obstetric care and to minimize errors in reporting. Respondents who had difficulty reporting the number of patients for a month timeframe were given the option to provide information for a typical year or for the past year. Typical and past month patient counts were averaged, and then multiplied by 12 to get an estimate for the calendar year. (Typical and past year patient counts were divided by 12, then averaged together, then multiplied by 12.)

#### Adjustment for complications from spontaneous abortion

Applying the weight to this data yields an estimate of the total number of abortion complications treated per year at all health facilities. As this number includes both spontaneous and induced abortion complications, we needed to exclude women with spontaneous abortions. It is difficult to differentiate between complications due to voluntary pregnancy interruption and those due to a spontaneous abortion because not only are the symptoms of the two often very similar, but stigma also prevents women from admitting to having sought an abortion. To address this issue, we used an indirect method to separate out spontaneous abortion complications from the estimated total number of complications treated in health facilities.

We assume that women who experience late spontaneous pregnancy loss (13–21 weeks’ gestation) are likely to require care at a health facility due to the higher frequency and severity of complications after the 13^th^ week of gestation [20]. About 2.89% of pregnancies recognized at six weeks’ gestation end in spontaneous abortion during weeks 13–21. As 84.575% of recognized pregnancies result in live births, the number of spontaneous abortions during weeks 13–21 is roughly equal to 3.417% of live births (2.89/84.575) [20,21]. An estimate for live births in Uganda in 2013 was calculated by applying the age-specific fertility rates from the 2011 UDHS (assumed to have remained fairly constant over the 2 year period) to the 2013 regional population estimates for women aged 15–49, by five-year age groups.

The AICM requires a further adjustment because not all women needing treatment for complications of late miscarriages will obtain care in a facility. We assumed that the proportion of women obtaining care in a facility for a late spontaneous pregnancy loss is the same as the proportion of women giving birth in a health facility. According to the 2011 UDHS, this represented 63% of women giving birth; we projected this value to 2013, based on the assumption that births assisted by a trained health worker would continue to increase, as was the case between the 2006 and 2011 UDHSs [15,22]. These cases were removed from the total number of abortion complications treated in a health facility.

#### Estimating the multiplier

Not all women who have induced abortions experience complications, and those that do, do not necessarily obtain treatment in a health facility. To account for these cases, a multiplier or inflation factor must be calculated and applied to the estimated number of induced abortion complications treated in health facilities in order to estimate the total number of women who had an induced abortion. In general, the safer abortion services are, the higher the multiplier will be, because a lower number of women will experience complications that require medical treatment. Likewise, the less safe abortion services are, the lower the multiplier because a larger number of women will have serious complications that will require medical treatment. The multiplier is also affected by women’s access to health facilities. Where facilities are easily accessible, the proportion of women with complications who receive treatment will be higher, implying that the multiplier will be lower. The reverse will be the case in poor or underserved areas.

Data needed to estimate the multiplier is drawn from the HPS. Respondents were asked to make a series of estimations:

1. the percent distribution of women obtaining abortions according to type of provider;
2. the proportion of women likely to experience complications requiring medical care;
3. the proportion of women with complications who were likely to obtain care from a health facility (S2 Appendix).

Provider types included doctors, clinical officers, nurses, trained/certified midwives, pharmacists, traditional birth attendants, and the women themselves. Because the conditions under which women obtain abortions vary by socioeconomic status and their place of residence, respondents were asked to answer the above questions for each of four subgroups of women: urban poor, urban nonpoor, rural poor and rural nonpoor. Poor and nonpoor were defined using women’s level of education, as data on income is not available in Uganda. Poor women were defined as those who had had seven or fewer years of schooling, and nonpoor women as those with eight or more years of schooling [15].

Based on these data, we estimated, for each of the four subgroups, the proportion of women treated for abortion complications among those who had an induced abortion. Percentages were weighted by the relative size of the subgroups to yield the proportion of all women who had an abortion that were treated for complications nationally and regionally. The multipliers are the inverse of these proportions.

The estimated proportion of women getting treatment for abortion complications was 29.7%, therefore the national estimated multiplier was 3.37 (100/29.7). Regional multipliers were calculated for the same four major regions the country was divided into during the 2003 study to facilitate comparison: Central, Eastern, Northern and Western. The regional multipliers were then applied to the regions that they represent currently. Thus, the multiplier estimated for the old Central region (3.46) served as the multipliers for Central 1, Central 2 and Kampala; the multiplier estimated for the Northern region (3.31) was applied for Karamoja, North and West Nile; the multiplier estimated for the Eastern region (3.87) served as the multipliers for East Central and Eastern; and the multiplier estimated for the Western region (3.27) was used for South West and Western. These multipliers were applied to the regional estimates of complications from induced abortions to calculate estimates for the total number of abortions in each region. There is a small difference in the total number of abortions when the national multiplier is applied to the national number of complications compared to the sum of the regional complications. We adjusted the regional totals of abortions slightly, such that they sum up to the total number of abortions obtained using the multiplier. National and regional confidence intervals were constructed around the estimates of induced abortion, based on the 95% confidence intervals around the estimate of abortion complications treated in a year at all health facilities.

#### Estimating unintended pregnancy

Using the estimates of abortion from this study, recent births data from the UDHS, and population data, we estimated the rates of pregnancy and pregnancy outcomes; specifically, the proportion of pregnancies ending as planned and unplanned births, abortions, and miscarriages.

To estimate numbers and rates of unintended pregnancies, we first calculated the number of unplanned births, using data from the 2011 UDHS. This was obtained by estimating the proportion of births during the five years prior to the survey that had been unplanned (i.e. mistimed or unwanted at the time of conception); this proportion was applied to the number of live births in 2013. Summing the numbers of unplanned births, induced abortions (estimated in this study), and miscarriages resulting from unintended pregnancies (calculated as 20% of unplanned births and 10% of abortions) [21,23] yielded estimates of the number of unintended pregnancies. With this number, we estimated the proportion of all pregnancies that were unintended and applied this number to the population of women aged 15–49 to obtain the rate of unintended pregnancies. In the 2003 study, unintended pregnancies ending as miscarriages were not included in the calculations of the unintended pregnancy rates. We have recalculated the unintended pregnancy rate for Uganda for 2003 to facilitate comparison with the 2013 data and allow for analysis of trends over the past decade.

## Results

### Provision of Post Abortion Care

Of the 2,274 facilities in Uganda with potential to provide PAC, about 89% are estimated to treat post abortion complications. Almost half of these facilities provide both inpatient and outpatient PAC services (49%). One fifth of the facilities (20%) provide only inpatient services, while another fifth (19%) provide only outpatient services. Eleven percent do not provide PAC at all (not shown).

Facilities that provide PAC are unevenly distributed across the country. The largest and the second largest number of facilities that treat abortion complications (335 and 265, respectively) were estimated to be in the Central 1 and Eastern regions, while Karamoja contains the least with 46. In all other regions, the estimated number of facilities providing PAC services varies between 129 and 262 (Table 2). Examining the availability of PAC by the number of beds per 100,000 women of reproductive age presents a completely different picture. West Nile, one of the poorest regions [24] has the highest estimated number of beds (853 per 100,000 women 15–49) whereas Kampala, arguably one of the most modern and urbanized regions, has the lowest availability (229 beds per 100,000 women 15–49, Table 2).

**Table 2:** Estimated indicators of PAC availability and service provision by facility type, nationally and regionally, 2013.

	Total	Central 1	Central 2	East Central	Eastern	Kampala	Karamoja	North	South West	West Nile	Western
**Availability of PAC (weighted data)**											
Total number of HF treating abortion complications	2,015	335	234	154	265	249	46	134	262	129	207
Total number of beds in HF treating abortion complications	39512	4740	3943	2346	5095	1813	1432	5019	6128	3946	5049
Number of post abortion facilities per 100,000 women 15–49	25	38	28	19	23	31	17	20	26	28	18
Number of beds in post-abortion facilities per 100,000 women 15–49	491	536	473	290	433	229	538	737	603	853	447
Abortion caseload per facility that offers PAC^*^											
Average annual number of abortion complications per facility											
All facilities	64	52	55	86	50	86	60	111	53	59	56
Hospitals	290	281	214	342	243	534	100	352	206	181	296
Health Centers Level IV	85	84	116	124	68	34	48	98	65	98	108
Health Centers Level III	49	49	39	72	34	93	56	91	30	45	36
Private midwives	36	36	37	35	6	37	0	0	60	0	18
Government sector facilities	75	75	61	112	52	927	61	105	45	56	59
Private sector facilities	42	36	37	37	8	52	0	24	59	0	28
NGO facilities	70	68	77	42	63	78	57	280	72	74	68

Source: HFS

*Includes spontaneous and induced abortion complications. Weighted data

The estimated average patient caseload *per site* is highest in North (111), Kampala (86), and East Central (86) than in the other regions (50–59, Table 2). Furthermore, Kampala hospitals have the largest annual caseload (534 cases per site) compared to all other hospitals in the country. HC IVs and HC IIIs treat a much lower number of cases, which is expected as they are lower capacity facilities. Public and NGO facilities treat comparable average numbers of women with abortion complications (75 and 70), while private facilities, which mostly consist of private midwives, treat an average of 42 abortion complication patients per site per year (Table 2).

### Abortion Morbidity

In 2013, an estimated 128,682 women were treated for spontaneous and induced abortion complications in Uganda, with 93,265 or 72% receiving treatment for complications related to induced abortion (Table 3). In four regions (West Nile, South West, Karamoja, Central 1), the estimated proportion of induced abortion complications is around the national average (71%-79%). In Western, Eastern, Central 2 and East Central, the proportion is lower (56%-65%), while in North and Kampala the proportion is well above the national average (81% and 84%).

**Table 3:** Measures for calculating the estimated number and rate of women treated for induced abortion complications, by region, Uganda, 2013.

			No. of women treated			% of all abortion complications that are due to induced abortions	Induced abortion complications treatment rate*
Region	No. of Women aged 15–49	No. of live births	For spontaneous or induced abortions	For miscarriages	For induced abortions		
**Total**	**8,051,002**	**1,644,425**	**128,682**	**35,417**	**93,265**	**72**	**12**
Kampala	793,243	100,316	21,406	3,340	18,066	84	23
Central 1	884,045	160,952	17,400	3,659	13,741	79	16
Central 2	833,660	178,002	12,880	4,604	8,276	64	10
East Central	807,713	184,885	13,190	4,585	8,605	65	11
Eastern	1,177,190	288,789	13,181	5,497	7,685	58	7
Karamoja	266,139	59,903	2,747	627	2,120	77	8
North	681,161	142,634	14,883	2,857	12,026	81	18
South West	1,015,888	197,999	13,742	2,966	10,777	78	11
West Nile	462,846	96,401	7,618	2,185	5,433	71	12
Western	1,129,117	234,543	11,635	5,098	6,537	56	6

Sources: HFS; Uganda DHS 2011; 2015 UN World Population Prospects data for 2013 (total female population 15–49)

* Number of induced abortions complications treated in health facilities per 1,000 women 15–49.

The estimated national rate of hospitalization for induced abortion–the number of women treated each year due to induced abortion complications per every 1,000 women aged 15–49, is 12. In Kampala this rate is substantially higher (23 per 1,000) than both the national average and any other region in the country (Table 3).

### Abortion Incidence

Applying the national multiplier (3.37) to the estimated total number of women receiving treatment for complications of induced abortion (93,265), we estimated that 314,304 induced abortions occurred in Uganda in 2013, with a range from 191,776 to 438,524 (Table 4). Induced abortion appears most prevalent in Kampala: Close to one fifth or 19% of all induced abortions occurred in this region. Each of the remaining nine regions accounted for 15% or less of all induced abortions (not shown).

**Table 4:** Estimated number of induced abortions, abortion rates and abortion ratios, by region Uganda 2013.

	Number of Induced Abortions			Abortion Rates‡			Abortion Ratios§		
Region	Low estimate*	Medium estimate†	High estimate*	Low estimate*	Medium estimate†	High estimate*	Low estimate*	Medium estimate†	High estimate*
**Total**	**191,776**	**314,304**	**438,524**	**24**	**39**	**55**	**12**	**19**	**27**
Kampala ^a^	9,302	60,797	112,234	12	77	142	9	61	115
Central 1	37,738	46,241	55,125	43	52	62	23	29	35
Central 2	20,619	27,851	35,282	25	33	42	12	16	20
East Central	20,818	32,391	44,153	26	40	55	11	18	25
Eastern	17,088	28,926	40,911	15	25	35	6	10	15
Karamoja	5,102	6,825	8,598	19	26	32	9	11	15
North	26,971	38,718	50,719	40	57	75	19	27	37
South West	27,324	34,275	41,500	27	34	41	14	17	22
West Nile	13,492	17,491	21,623	29	38	47	14	18	23
Western	13,322	20,790	28,379	12	18	25	6	9	13

Sources: HFS; HPS; Uganda DHS 2011, 2015 UN World Population Prospects data for 2013 (total female population 15–49)

* Low and high estimates are based on 95% confidence intervals of the medium estimate

† Medium estimates of abortions were derived from the HPS as described in the methods section.

‡ The abortion rate is the number of abortions per 1,000 women aged 15–49

§ The abortion ratio is the number of abortions per 100 live births

^a^ The large confidence intervals for Kampala reflects the high proportion of PAC reported by hospitals in this region

The estimated induced abortion rate for the country is 39 per 1,000 women aged 15–49, with a range of 24 per 1,000 to 55 per 1,000. Regional variation in abortion rates is very large between Kampala and the remaining regions. Kampala’s estimated abortion rate is 77 per 1,000, twice the national average, with the next highest rates found in Central 1 and North (52 and 57 per 1,000, respectively). The abortion rates in Central 2, South West, West Nile and East Central regions are similar or close to the national average (33 to 40 per 1,000). In Western, Eastern, and Karamoja, the abortion rates are lower than the national average (18–26 per 1,000), exhibiting large differences from the national rate, and the other individual regional rates.

Another way to consider induced abortion is examining it as a ratio with respect to the number of live births. This can serve as an indicator of women’s motivation to avoid carrying an unwanted pregnancy to term. At the national level, 19 (CI = 12–27) abortions are estimated for every 100 live births. An abortion ratio of 19 indicates that there is one abortion for every five live births (Table 4). The abortion ratio varies across regions, but most regions fall between 9 and 29 abortions per 100 live births. In Kampala however, the abortion ratio is nearly three times the national ratio, at 61 abortions per 100 live births.

#### Trends in abortion incidence

Uganda has one of the highest rates of population growth in the world. Its total population grew from 17.38 million in 1990 to 39.03 million in 2015 [19]. Between 2003 and 2013, the total Ugandan female population aged 15–49 increased 40%, ─from 5.7 million to 8.0 million (Table 5). Live births increased 27%, from an estimated 1.3 million to 1.6 million and the total number of pregnancies increased 24%, from an estimated 1.8 million to 2.3 million. Over the ten-year period, the total fertility rate decreased 10% while the unwanted fertility rate decreased 15%.

**Table 5:** National trends in estimated abortion incidence and related measures: 2003–2013 and 2000/01-2011.

**Measure**	**2003 (Revised)** *	**2013**	**% change**
No. of women aged 15–49	5,769,000	8,051,002	40
No. of live births	1,295,409	1,644,424	27
No. of pregnancies	1,877,673	2,319,043	24
Total fertility rate	6.9	6.2	-10
Total wanted fertility rate	5.3	4.5	-15
**No. of women treated in health facilities**			
For any abortion	109,926	128,682	17
For spontaneous abortion	25,982	35,417	36
For induced abortion	83,944	93,265	11
**Measures of abortion**			
No. of induced abortions	293,804	314,304	7
Abortion rate†	51	39	-24
Abortion ratio‡	16	14	-13
Induced abortion complications treatment rate§	15	12	-20
**Antecedents on abortion**	**2000/01**	**2011**	**% change**
**Contraceptive use/unmet need**			
*Among married women aged 15–49*			
% using any method	23	30	30
% using traditional method (periodic abstinence and withdrawal)	9	4	-55
% using modern method¥	14	26	86
% with unmet need for modern contraception**	51	38	-26
*Among unmarried sexually active women aged 15–49*			
% using any method	44	43	-2
% using traditional method (periodic abstinence and withdrawal)	6	5	-17
% using modern methods¥	38	38	0
% with unmet need for modern contraception**	45	45	0
**Unplanned fertility**			
% of births unplanned (mistimed + unwanted)	38	44	16
% of births mistimed	24	32	33
% of births unwanted	14	12	-14

Sources: HFS, HPS, Uganda DHS 2000/01, Uganda DHS 2011, 2015 UN World Population Prospects data for 2003 and 2013 (total female population 15–49)

*All data for 2003 was recalculated to use updated population data.

† The abortion rate is the number of abortions per 1,000 women aged 15–49.

‡ The abortion ratio is the number of abortions per 100 pregnancies.

§ The abortion treatment rate is the number of induced abortion complications treated in health facilities per 1,000 women 15–49.

¥ Includes pill, IUD, injectables, implants, spermicides, condoms, and female and male sterilization.

** Women have an unmet need for modern contraception if they want no more children or do not want a child in the next two years, are married or unmarried and currently sexually active and are using traditional method of contraception (periodic abstinence or withdrawal) or no method at all.

The estimated number of induced abortions in Uganda increased 7%, from 293,804 in 2003 to 314,304 in 2013. The estimated abortion rate decreased by 24%, from 51 to 39 per 1,000, while the abortion ratio decreased by 13% from 16 induced abortions per 100 pregnancies in 2003 to 14 per 100 pregnancies in 2013 (Table 5). These rates suggest a declining trend in abortion behavior among Ugandan women between 2003 and 2013.

Contraceptive use has increased over the past decade. In 2011, 30% of married women used a method of family planning, up from 23% in 2000/01 (Table 5). A more noticeable increase occurred in the use of modern contraceptive methods, where use among married women increased from 14% to 26% over the ten-year period. Despite this increase, the proportion of married women with unmet need for contraception barely changed, only dropping slightly from 40% to 38%. Among unmarried sexually active women, unmet need has stagnated at the high level of 45%. Overall, the percentage of women with unmet need fell just 0.2 percentage points per year during the decade of 2000/01-2011.

#### Unintended pregnancy

There were an estimated 2,319,043 pregnancies to women aged 15–49 in Uganda in 2013 (Table 6), with an overall pregnancy rate of 288 pregnancies per 1,000 women. This rate was lowest in Kampala (236 per 1,000) and highest in Eastern (321per 1,000). The estimated national unintended pregnancy rate was149 per 1,000 women aged 15–49. Regional estimates vary widely: Karamoja has the lowest rate with 61 per 1,000 women, while West Nile, Eastern, East Central and North exhibit the highest rates (between 164 and 198 per 1,000 women). The remaining regions all have rates closer to the national average (between 130 and 153 per 1,000 women).

**Table 6:** Estimated pregnancies, pregnancy rates, and distribution of pregnancies by outcome, nationally and regionally, Uganda 2013.

Regions	No. of pregnancies	Pregnancy rate*	Unintended pregnancy rate†	% pregnancies that are unintended‡	% of pregnancies ending in				
					Planned births	Planned pregnancies ending in miscarriages	Unplanned births	Abortion	Unintended pregnancies ending in miscarriages
**Total**	**2,331,681**	**288**	**149**	**52**	**40**	**8**	**31**	**14**	**8**
Kampala	204,659	236	132	56	37	7	17	33	7
Central 1	243,379	276	138	50	42	8	24	19	7
Central 2	242,202	293	153	52	40	8	33	11	8
East Central	265,081	319	172	54	38	8	34	13	8
Eastern	374,731	321	175	55	38	8	39	8	9
Karamoja	82,253	298	61	20	66	13	9	9	3
North	201,693	314	198	63	31	6	36	18	9
South West	271,200	271	121	45	46	9	26	12	6
West Nile	139,325	292	164	56	37	7	35	13	8
Western	307,158	270	130	48	43	9	34	7	8

Sources: HFS, HPS, Uganda DHS 2011, 2015 UN World Population Prospects data for 2003 and 2013 (total female population 15–49). Notes: We assume that the age specific fertility rates (ASFR) and the wantedness status of births based on the 2011 UDHS applies to 2013.

* The pregnancy rate is the number of pregnancies (live births + abortions + miscarriages) per 1,000 women aged 15–49 per year.

†Number of unintended pregnancies (unplanned births + abortions + unintended pregnancies ending as miscarriages) per 1,000 women aged 15–49 per year.

‡The percent of pregnancies that are unintended is the number of unintended pregnancies divided by the total number of pregnancies per 100 women

In 2013, an estimated 52% of pregnancies to Ugandan women were unplanned (Table 6). Regionally, this proportion ranges from as low as 20% in Karamoja to as high as 63% in the North region. Unplanned births are estimated to represent about one third of all pregnancies, while abortions make up 14%. The proportion of unintended pregnancies ending in abortion was lowest in Eastern (8%) and Western (7%) and very high in Kampala (33%). In all other regions, the proportion of pregnancies ending in abortion ranged between 9–19%.

### Limitations

Measuring abortion incidence in highly restrictive environments is a difficult task for researchers. As a result, one of the most plausible approaches to collecting this information is via the use of indirect methodologies. The AICM, like all other indirect estimation techniques, has limitations which have been discussed in previous studies that have used this methodology [14]. A main limitation of this approach is that the information for some key input variables depends on respondents’ perceptions. Official records in many countries where the abortion law is restrictive are often outdated, incomplete, or nonexistent. As a result, health care practitioners involved in PAC on a regular basis are often the best source of an accurate estimate of the number of patients that come to facilities with complications. In an incidence study carried out in Colombia [25] that used the AICM, official records were also requested in all sampled facilities. This exercise illustrated that in a country with a restrictive abortion law, not only is data on abortion very incomplete and inadequate for use in estimating national incidence of abortion, but also that HFS estimates can serve as an approximate for actual facility counts when they are unavailable. The current study also relied on perceptions from the HPS to calculate the multiplier. Although selection of these respondents were based on criteria such as knowledge and experience with abortion provision in both urban and rural areas of the country, the estimated multiplier should be considered as an approximate measure. Therefore, the AICM is a technique that can only estimate the incidence of abortion, not provide an exact figure.

## Discussion

We estimate that every year 93,265 women are hospitalized for treatment of complications from induced abortion. This is equivalent to an annual rate of 12 per 1,000 women aged 15–49. Although this rate has declined from 15 per 1,000 in 2003, morbidity due to unsafe abortion is still prevalent in Uganda, and is actually higher compared to nearby countries such as Rwanda (7 per 1,000 women aged 15–44) [26], Kenya (9 per 1,000 women aged 15–49) and Nigeria (9 per 1,000 women aged 15–49) [27]. High levels of abortion morbidity have serious consequences not only on the health and life of women, but also on their finances and children’s emotional health and wellbeing. In a recent study among 1,338 women treated in 27 health facilities for complications of unsafe abortion, 73% of women reported loss of productivity and 60% reported that their children received less food than usual, missed school or both. Length of hospital stay was positively associated with higher odds of their children experiencing these negative consequences [28].

Kampala has the highest estimated rate of treatment for induced abortion (23 per 1,000 women 15–49). This high rate is to some extent expected. As the most urban and developed region of the country, Kampala has better conditions of and better access to health care [29]. A common complication of induced abortion is hemorrhage, and the ability to access emergency blood transfusion services in Kampala is a further incentive to patients to obtain (and for providers to refer to) services there [14,29]. Both of Uganda’s national referral hospitals are located in this region, one of which is also the largest tertiary level, public health care facility in the country. In fact, public facilities in this region reported more than 10 times the national average of treatment for abortion complications. Along with the quality of care, women may also be financially motivated to visit public facilities in Kampala, as women who receive PAC treatment in public facilities generally incur lower health care expenses than those treated in private or nonprofit facilities [28].

Although the estimated rate of treatment of complications is still high, the observed decline is likely the result of the large increase in the use of modern contraceptives and possibly, the use of safer methods to induce abortion such as manual vacuum aspiration (MVA) and misoprostol. Misoprostol was reported as the second most common method used by providers (10% in urban areas and 7% in rural areas, not shown). In the 2003 HPS, the most common method reported as used by physicians in both urban and rural environments was dilation and curettage (D&C), while catheters and oral drugs were reported as most commonly used by non-physicians [30]. In comparison, respondents of the 2013 HPS reported that physicians most commonly used MVA (27% in urban area and 23% in rural area), while oral and vaginal herbs were most commonly used among non-physicians (65% urban and 76% rural). Although the extent to which women use misoprostol and how well they use it is currently unknown, more than two thirds of health professionals interviewed in the HPS indicated that misoprostol has been available in the country as far back as 2008. In fact, distribution of misoprostol use for in safe abortion care first took place in 2009, though guidelines for its use in this setting were not established until 2010. Support for misoprostol use in PAC treatment would not occur until 2012; as a result, the impact of this increased availability still remains to be seen [31].

The treatment rate reveals only those cases of abortion complications that require and obtain medical care. There are other women who experience abortion complications that, despite requiring treatment in a health facility, do not seek or get care. According to health professionals interviewed in the HPS, this encompasses about 46% of Ugandan rural women and 22% of urban women.

The government has made efforts to improve reproductive health care. One initiative focused on improving PAC by training a large number of health care providers in the use of MVA [32]. However, access to this service is still limited. Eleven percent of facilities sampled in the HFS reported no provision of PAC. Among providers that currently use MVA, which include midwives and medical officers, the service is typically only offered in the daytime on working days. Additionally, more private midwives are trained in the use of MVA than those midwives who work at public facilities [33]. However, as the average number of PAC cases treated by private midwives is much lower than the caseload treated in public and NGO facilities, it would suggest that women seeking PAC are less likely to pursue services from private midwives when public or NGO services are available, or that these women have difficulty accessing private midwives.

Among facilities that do provide PAC, an estimated 14% provide counseling on contraceptives to some but not all patients who come in for PAC; 3% do not provide any counseling at all and 8% do not provide any contraceptive methods. This information and supplies could be essential in helping women better protect themselves against unintended pregnancy, thus reducing the need for future abortions. Reasons given by the HFS respondents for not providing post abortion counseling include lack of training in this area, lack of available/trained staff in health facilities, and the belief that post abortion counseling is only for those patients with induced abortions, and not for those presenting with other conditions related to abortion complications.

The estimated 314,304 induced abortions in 2013, which translates into 39 abortions per 1,000 women of reproductive age, is a large decline from 2003, when the estimated rate was 51 per 1,000 women. This decline may be plausible considering the impressive increases in: the use of modern contraceptive methods among married women (from 14% to 26%); the number of women of reproductive age (40% change); and unplanned childbearing (from 38% to 43%) (Table 5). Most of this increase has been due to mistimed births, births that occur much sooner than desired. The proportion of these births rose from 24% to 32% over the period 2000/01-2011, while the proportion of births not wanted at all only decreased slightly, from 14% to 12%. (Table 5).

The high estimated abortion rates of Kampala, North and Central 1 stem from different factors that are intrinsic to the regions. The exceptionally high rate of abortion for Kampala is most likely because women are crossing boundaries from neighboring districts to get access to the abortion procedure and/or access to treatment for abortion complications, a function of the better access and quality of health care providers available [29]. The high abortion rate estimated for Central 1 and North regions is likely associated with the fact that women are not meeting their fertility goals. Women from these regions have one to two more children than the number desired, while one in three women in Central 1 and almost one in two women in North have an unmet need for contraception. North region is also possibly influenced by the influx of refugees from the Democratic Republic of Congo (DRC); this region likely lacks the resources to meet the health care needs required by this type of population.

The moderately high abortion rates estimated for Central 2, South West, West Nile and East Central (33, 34, 38, and 40 per 1,000, respectively) are also likely associated with the fact that women are not fulfilling their reproductive needs. Contraceptive use in these regions is low (between 14%-29%) and unmet need for modern contraception is high (37%-44%). In East Central the gap between actual and wanted fertility is the largest among all regions (6.9 vs. 4.4). Women from this region are having almost three more children than they want, use of modern contraceptives is not high (28%), and both the proportion of women with an unmet need for contraception (46%) and the estimated unintended pregnancy rate (180 per 1,000) are the highest among all regions. In West Nile, women are having almost two more children than they desire (6.8 vs. 5.1), and contraceptive use is one of the lowest in the country (14%). Meanwhile, the level of unmet need for contraception (44%) and the estimated unintended pregnancy rate (164 per 1,000) are the second and fourth highest rates among all regions (Table 7). Estimates for West Nile might also need to be considered with respect to the refugees from Sudan. The majority (80%) of the displaced population that has settled in these areas consists of women and children, who have also been the main targets of kidnapping and sexual violence. As in North region, many basic needs, including health services, remain largely unfulfilled in these areas [34].

**Table 7:** Selected indicators of reproductive behavior of Ugandan women aged 15–49, by region, Uganda 2013.

	Total fertility rate (TFR)*	Wanted fertility rate (TWFR) †	Excess fertility‡	Estimated unintended pregnancy rate§	% using modern contraceptive method¥	% with unmet need for modern contraception**
Regions						
**Total**	**6.2**	**4.5**	**1.7**	**149**	**26**	**38.4**
Kampala	3.3	2.9	0.4	132	40.2	24.6
Central 1	5.6	4.2	1.4	138	30.5	33.3
Central 2	6.3	4.6	1.7	153	29.6	39.5
East Central	6.9	4.4	2.5	172	27.7	46.2
Eastern	7.5	5.3	2.2	175	23.2	41.3
Karamoja	6.4	5.8	0.6	61	7.4	21.0
North	6.3	4.3	2.0	198	23.3	43.2
South West	6.2	4.4	1.8	121	25.1	41.3
West Nile	6.8	5.1	1.7	164	13.6	43.9
Western	6.4	4.7	1.7	130	26.6	36.5

Source: HFS, HPS, Uganda DHS 2011

* Total fertility rate is the total number of live births a woman would have had if she was subject to the current age-specific fertility rates throughout the reproductive ages (15–49 years).

† Wanted fertility is the total number of live births a woman would have had if all unwanted births had been avoided.

‡ Excess fertility is the number of children a woman has vs. the ideal number of children she desired.

§ Number of unintended pregnancies (unplanned births + abortions + unintended pregnancies ending as miscarriages) per 1,000 women aged 15–49 per year.

¥ Includes pill, IUD, injectables, implants, spermicides, condoms, and female and male sterilization.

** Women have an unmet need for modern contraception if they want no more children or do not want a child in the next two years, are married or unmarried and currently sexually active and are using a traditional method of contraception (periodic abstinence or withdrawal) or no method at all.

Furthermore, activity along the Trans Africa Highways, which run though Western, South West, Central 2, Central 1, Kampala and East Central, might also play a role in the observed abortion rate estimates. There is evidence of a high frequency of transactional sex taking place between male drivers and female commercial sex workers, with some women reporting more than 20 unique partners per month, and as much as 63 sexual encounters in the same time frame [35,36]. As sex workers report nonuse of condoms up to 20% of the time, as well as inconsistencies in their hormonal method use, these women are particularly vulnerable to unintended pregnancies, compounded by being in areas with limited availability of health facilities and where women face stigma from providers when they do try to access health care [35–38].

Eastern, Western and Karamoja regions have low abortion rate estimates (18–25 abortions per 1000 women aged 15–49). This finding is likely due to a combined effect of high fertility and an inability to prevent unintended pregnancy. Around 50%-55% of the pregnancies in Western and Eastern regions are unintended. Furthermore, only around a quarter of women from these two regions use a modern method for contraception and 37–41% of them have an unmet need for modern methods (Table 7). These factors, in addition to the high excess fertility (around two children per woman) and the high proportion of unplanned births (34–39%), suggest that women in these regions are more likely to carry an unintended pregnancy to term than to resolve it with an abortion. Although the estimated abortion rate in Karamoja is similar to that estimated in Western region, reproductive indicators are much lower in Karamoja: just 20% of the pregnancies are unintended; only 7% of women use a modern contraceptive; unmet need for modern contraception is 21% and excess fertility is the lowest in the country (only 0.6). All three of these regions have the lowest proportion of estimated pregnancies ending in abortion (7%-9%).

Most induced abortions are the result of unintended pregnancy. Findings from this study indicate that unintended pregnancy is still very high in Uganda; the estimated rate has hardly changed over the past decade: from 158 per 1,000 women aged 15–49 in 2003 to 149 per 1,000 in 2013. The latter rate is three times higher than the rate for the world (55 per 1,000 women aged 15–44) [39], and much higher than the estimated rates for Eastern Africa (118 per 1,000 women aged 15–44) [40], Ethiopia (101 per 1,000 women aged 15–44) [41] and Rwanda (114 per 1,000 women aged 15–44) [42].

The reason for this continued, high level of unintended pregnancy reflects contraceptive patterns exhibited by Ugandan women. Despite near universal knowledge of contraceptive methods, only about a quarter of married woman report current use of a modern contraceptive method, and about half of unmarried women [15]. Among these users, most married women report using injectables (as their most effective method), while injectables and male condoms are most reported by unmarried women. Still, of all Ugandan family planning users, 43% discontinue use of a method within the first year of use. In fact, the injectable, the most commonly used effective method among contraceptive users, has the second highest discontinuation rate, with fear of side effects cited most often as the reason for cessation of use [15]. Meanwhile, male condoms not only have a lower typical-use effectiveness than other modern methods, but also have the fourth highest discontinuation rate, most often due to relationship-based reasons (infrequent sex, partner away, dissolution) [15,43].

Unmet need for modern contraception has also remained nearly unchanged and at a high level during the past decade. According to the 2011 UDHS, the proportion of married women having an unmet need for modern contraception—that is, those women who either do not want a child in the next two years or do not want any (more) children, and are using a traditional method of contraception (periodic abstinence, withdrawal) or none at all—was 38%, a proportion that is lower than ten years ago (51%) [5], yet still high. Moreover, the level of unmet need among sexually active unmarried women has not changed at all and is currently greater than that of married women—45%. Despite improvement among married women, levels of unmet need for modern contraception in Uganda are still some of the highest in Sub-Saharan Africa [40].

**Implications for policies and programs.** This study has several implications for abortion and contraceptive services in Uganda. The increase in the proportion of pregnancies that are unintended, the high proportion of women with unmet need for modern contraception and the extremely high population growth rate, strongly suggest that more efforts are needed to improve family planning services and PAC. Many of the reasons health care providers reported for not providing counseling and contraceptives to PAC patients reflect a lack of prioritization of these services. Women receiving PAC services are most in need of protection against an unintended pregnancy because they can resume their fertility much sooner compared to women who give birth. Health authorities should allocate greater resources for PAC, including supplying relevant kits, encouraging use of modern, more effective and less invasive methods, particularly MVA and misoprostol and training health service staff [44].

Contraceptive counseling also needs to be strengthened. Nonuse of modern contraception typically accounts for the vast majority of unintended pregnancy. Ineffective use of modern contraceptives and underuse of effective, long acting methods accounts for the remainder of unintended pregnancies [15,45,46]. For example, long acting reversible contraceptive methods (LARCs), which have efficacy rates ranging between 94 and 99%, are used by slightly over half of the women who use contraception [15,43]. Previous work on contraceptive use in Uganda indicates that misperceptions regarding their mechanisms, concern over side effects, lack of supplies, attitudes about partner involvement and provider bias all potentially contribute to whether a woman initiates LARC use [47–49]. To encourage method uptake and correct and consistent method use, contraceptive counseling should acknowledge the concerns women have about methods, and incorporate efforts to mitigate the high discontinuation rates during the first year of method use. This can be done by acknowledging the challenges women and couples experience around initiating and maintaining method use. Facilities also need to be better equipped to provide women with the methods they desire, and training around contraceptive counseling should consider challenging the potential biases of providers.

Coverage also needs to be expanded to reach the women with post abortion complications who experience difficulty accessing facilities and obtaining medical care. According to health professionals interviewed in the HPS, about half of poor women and about one fifth of non-poor women in Uganda do not get treatment in a medical facility for post abortion complications. Additionally, the majority of these professionals believe that stigma and fear of mistreatment at health care facilities is a primary barrier for women seeking PAC services. Awareness of this issue should be incorporated into the training of health care providers.

Finally, since the law and policies governing abortion lack clarity and consistency, it is difficult for providers and women to know what to do when faced with an abortion decision, leaving women to consider obtaining unsafe alternatives. In service of helping women avoid risking their health and lives, the laws and policies around abortion should be better clarified and there should be efforts to increase awareness of these policies among the medical community, the judiciary system and the women themselves. Health authorities should also ensure that safe services are available and affordable, that health facilities are equipped appropriately, and that health care providers are trained to provide adequate quality of care.

## Supporting Information

S1 Appendix Health Facilities Survey. (PDF)

S2 Appendix Health Professional Survey. (PDF)
