# Supplementary material for: Incidence of Induced Abortion in Uganda, 2013: New Estimates Since 2003
Source: PLoS One. 2016 Nov 1;11(11):e0165812. doi: 10.1371/journal.pone.0165812 (PMC5089684; doi:10.1371/journal.pone.0165812)
Supplement: S1 Appendix — (PDF) [file pone.0165812.s001.pdf]

Qr. # | | |

STUDY ON ABORTION AND POSTABORTION CARE

SURVEY OF HEALTH FACILITIES IN UGANDA, 2013

CONSENT

My name is_____________. and I am working with the Makerere University School of Public Health. The School in conjuction with the Guttmacher Institute, a USA research organization, is conducting a national study to assess post-abortion care. We would like to ask for your cooperation in getting a better picture of the situation in Uganda. The research will provide information about reproductive health care and will contribute to the improvement of maternal health in Uganda and beyond.

Your health facility has been randomly selected to be part of this study. Your responses about care provided in your facility will be completely confidential and will be used for research purposes only. No personal reference will be made to your participation in this study. Participating in this survey poses little if any risk. Your responses and those of other health providers in Uganda will be used to describe the general picture of abortion care in Uganda. This interview will take at least an hour of your time.

While we would like to note that your expertise and experience make your participation critical and your views valuable to us, we would also like to emphasize that your participation is absolutely voluntary and that you are at liberty to terminate the interview at any time. Although we expect that the results of this study will help improve the nation's health care system, there will be no direct financial benefit to you personally.

Do you agree to proceed with the interview?

AGREE………………………………. ☐

DO NOT AGREE…………………… ☐

Reason for refusal:____

____

____

Thank you

Health Facility Information

001 HEALTH FACILITY NAME: ________________________ |__|__|__|

002 PHYSICAL ADDRESS: ________________________

003 COUNTY: ________________________ |__|__|

004 DISTRICT: ________________________ |__|__|

005 LOCATION:
- URBAN ………………………….. 1
- RURAL ………………………….. 2

006 TYPE OF HEALTH FACILITY
- HOSPITAL 1
- HEALTH CENTER IV 2
- HEALTH CENTER III 3
- PRIVATE MIDWIFE 4

007 OWNERSHIP
- Public/Government…………………………………………. 1
- Non-governmental organization (NGO)…………………. 2
- Private…………………………………………………… 3

008 INTERVIEWER'S NAME: ________________________

009 INTERVIEWER'S CODE: |__|__|

010 TIME STARTED: |__|__| hr |__|__| min TIME ENDED: |__|__| hr |__|__| min

011 DATE OF INTERVIEW: |D|D| |M|M| |Y|Y|Y|Y|

012 INTERVIEW OUTCOME:
- Completed………………………………………… 1
- Refused…………………………………………… 2
- Incomplete………………………………………… 3
- Other (specify) ________________ 96

Please specify why the interview is not complete ________________________

____

Interviewer observations: ________________________

____

____

Module I: Background Information

	Questions and Instructions	Responses, Codes and Filters
101	What is your official position [profession] at this facility?<br><br>***[Interviewer: DO NOT PROMPT.<br>Circle the category that applies to the respondent.]***<br><br>***Nurse = Enrolled and registered nurses<br>Trained midwife = Enrolled and registered midwives<br>Comprehensive nurse = Enrolled and registered comprehensive nurses***	OB-GYN…… 1<br>Medical Officer/GP…… 2<br>Clinical Officer…… 3<br>Trained Midwife…… 4<br>Nurse…… 5<br>Comprehensive nurse …… 6<br>Nurse Aide…… 7<br>Other (Specify) ________ 96
102	Respondent's sex	Male…… 1<br>Female…… 2
103	How old are you?	[ ][ ] Years
104	What is your highest professional/academic qualification you have completed?<br><br>***[Interviewer: Read the categories if necessary.]***	Certificate…… 1<br>Diploma …… 2<br>Degree…… 3<br>Post graduate…… 4<br>Other (Specify) ________ 96
105	Whats your main responsibility at this facility?<br><br>***[Interviewer: Read the categories if necessary.]***	Facility administrator…… 1<br>Facility in-charge…… 2<br>Unit/Department in-charge…… 3<br>Health care provider (non-administrative)…… 4<br>Other (Specify)________ 96
106	How many years have you worked in your current primary profession?<br>***[Interviewer: If less than 1 year, fill 00]***	[ ][ ] Years
107	How many years have you worked in your primary profession IN THIS FACILITY?<br>***[Interviewer: If less than 1 year, fill 00]***	[ ][ ] Years
108	Which units/departments does this facility have?<br><br>***[Interviewer: READ out all categories. Multiple responses are allowed]***	Outpatient (ambulatory)…… 1<br>Inpatient unit…… 1<br>Operating room/theater…… 1<br>Evacuation room…… 1<br>Gynaecological Ward…… 1<br>Labor Ward…… 1<br>Maternity Ward…… 1<br>MCH/FP unit…… 1<br>Intensive care unit (ICU)…… 1<br>Emergency ward…… 1<br>Laboratory…… 1<br>Pharmacy…… 1<br>Other (specify) ________ 96<br>________

109	Which of the following women's reproductive health services does this facility provide?<br><br>***[Interviewer: Read out all categories. Multiple responses are allowed]***	OB-GYN specialists/consultants…………………. 1<br>Maternity/antenatal care…………………………. 1<br>Delivery services... …………………..……. 1<br>Post abortion care…..…………………………. 1<br>Post-abortion contraceptive counselling………. 1<br>HIV/AIDS (Diagnosis and/or treatment) ………. 1<br>Inmunization …………………………………. 1<br>None of the above……………………………… 1<br>Other (specify) ______________ 96
110	How many beds does this facility have in total?	☐☐☐☐ Beds
111	How many people in this facility are trained to provide PAC services?	☐☐ Staff **[If '0' Skip to Module 2]**
112	Is a staff member trained in PAC available to attend to patients rarely, sometimes, or always?<br><br>***[If respondent notes that staff members have different availability, take the answer for the staff member that is MOST available.]***	Rarely………………………… 1<br>Sometimes…………………… 2<br>Always……………………… 3

Module II: Postabortion Care

Now, I would like to ask you some questions regarding **medical care for abortion patients treated at this facility, regardless of whether the abortion was spontaneous or induced.** I will also ask about **the number of patients that are treated for abortion complications at this facility.** By abortion complications, we are referring to those complications that are severe enough to **need treatment** in a health facility. Abortion complications include not only **extremely serious cases** such as sepsis or a perforated uterus, but also those cases which are termed "incomplete abortions." Incomplete abortions are usually identified by heavy bleeding, and present a somewhat **less severe** health risk to the woman, but still need treatment at a health facility. In answering the following set of questions concerning abortion complications, please keep this definition in mind.

[Interviewer: Note that the abortion complication questions refer to both spontaneous and induced abortions. You should repeat this as often as possible while completing this section.]

201	a. Normally (Generally), about how many deliveries take place at your facility each month?<br><br>***[Interviewer: Ask for the total number in the year 2012, in case respondent can't answer per month]***<br><br>b. Out of this number, about how many are still births?	[1] Deliveries per month [ ][ ][ ][ ]<br>**OR**<br>[2] Total number of deliveries during the **2012** calendar year [ ][ ][ ][ ]<br><br>[ ][ ][ ]<br>***[If no still births are represented, write in '0.']***
202	a. Does this facility treat women with spontaneous or induced abortion?<br><br>b. In which sections of this health facility are post-abortion patients treated?<br><br>***[Interviewer: Read out the list of wards and circle all that apply]***	Yes………… 1 → **[Go to Q202 part b.]**<br>No………….. 2 → **[Go to Q208]**<br><br>Outpatient ward/Recovery room…………………… 1<br>Operating Room……………………………….. 1<br>Evacuation Room…………………………………… 1<br>Gyneacological ward……………………………… 1<br>Labor Ward…………………………………………. 1<br>Maternity ward……………………………………… 1<br>General female ward……………………………… 1<br>Intensive care unit (ICU)…………………………… 1<br>Emergency room…………………………………… 1<br>Other (specify) ____________ 96
203	In this facility, are **post-abortion care patients** treated as **outpatients** (they don't spend the night in the facility), as **inpatients** (they spend at least one night in the facility) or both?	Outpatient only .... 1<br>Inpatient only ……………….. 2 → **[Skip to Q206]**<br>Both…………………………… 3
204	During **a typical** month, about how many such **post-abortion** care **patients** would you estimate are treated as **outpatients** at this facility as a whole? Please remember to include all **post-abortion care patients** whether they are due to spontaneous or induced abortions.<br><br>***[Interviewer: probe to elicit a response for a typical month; if respondent is not able to provide you with that estimate, then probe for the number of outpatients in a typical year. Specify that this is a full calendar year (i.e. from January to December). Please reiterate to the respondent that the number is both spontaneous and induced abortion patients and should take into consideration all wards of the facility. If a range is given, probe further for an average number]***	[1] Number of outpatients in a **typical** month<br>[ ][ ][ ][ ][ ]<br>**OR**<br>[2] Number of outpatients in a **typical** year<br>[ ][ ][ ][ ][ ]

205	In the **past** month, about how many **post-abortion care** patients were treated as **outpatients** in this facility as a whole (please remember to include all **post-abortion care patients**, whether they are due to spontaneous or induced abortions)<br><br>***[Interviewer: Probe to elicit a response for the past month; if respondent is not able to provide you with that estimate then probe for the number of outpatients during the year 2012. Specify that this is a full calendar year (i.e. from Jan to Dec). Please reiterate to the respondent that the number should take into consideration all wards of the facility. If a range is given, probe further for an average number]***	1 Number of **outpatients** in the **past** month<br>☐☐☐☐☐<br><br>**OR**<br><br>2 Number of **outpatients** in the **past** year **2012**<br>☐☐☐☐☐
[IF **Q203** INDICATES THAT **INPATIENT SERVICES ARE PROVIDED**, THEN ANSWER **Q206-207** IF NOT, **GO TO Q208**]		
206	During **a typical** month, about how many such **post-abortion care patients** would you estimate are treated as **inpatients** at this facility as a whole? Please remember to include all **post-abortion care patients** whether they are due to spontaneous or induced abortions<br><br>***[Please probe to elicit a response for a typical month; if respondent is not able to provide you with that estimate then probe for the number of inpatient on typical year. Specify that this is a full calendar year (from Jan to Dec). Please reiterate to the respondent that the number is for spontaneous and induced abortion patients, and should in to consideration all wards of the facility. If a range is given, probe further for an average number]***	1 Number of **inpatients** in an a **typical** month<br>☐☐☐☐☐<br><br>**OR**<br><br>2 Number of **inpatients** in a **typical** year.<br>☐☐☐☐☐
207	In the **past** month, about how many **post-abortion care patients** were treated as **inpatients** in this facility as a whole? Please remember to include all **post-abortion patients**, whether they are due to spontaneous or induced abortions<br><br>***[Interviewer: Please probe to elicit a response for the past month; if respondent is not able to provide you with that estimate, then probe for the number of inpatients during the year 2012. Please reiterate to the respondents that the number should take consideration all wards of the facility. If a range is given, probe further for an average number]***	1 Number of **inpatients** in the **past** month<br>☐☐☐☐☐<br><br>**OR**<br><br>2 Number of **inpatients** in the **past** year (**2012**)<br>☐☐☐☐☐
[IF THE RESPONSE FOR Q202A IS **"NO,"** **ONLY ASK Q208, Q209 AND Q210, THEN SKIP TO MODULE 4.]**		
208	During **a typical** month, about how many **post-abortion care patients** would you estimate are **referred** from this facility as a whole? Please remember to include all **post-abortion care patients**, whether they are due to spontaneous or induced abortion.<br><br>***[Probe to elicit a response for a typical month; if respondent is not able to provide you with that estimate, then probe for the number of referrals in a typical year. Specify that this is a full calendar year (Jan-Dec). Please reiterate to the respondent that the number is for spontaneous and induced abortion patients, and should take into consideration all wards of the facility. If a range is given, probe further for an average number]***	1 Number of **referrals** in an **typical** month<br>☐☐☐☐☐<br><br>**OR**<br><br>2 Number of **referrals** in a **typical** year (**2012**)<br>☐☐☐☐☐

209	In the **past** month, about how many **post-abortion care patients were referred** from this facility as a whole? Please remember to include all **post-abortion care patients**, whether they are due to complications of spontaneous or induced abortions.<br><br>***[Please probe to elicit a response for the past month; if respondent is not able to provide you with that estimate, then probe for the number of referral during the year 2012. Please reiterate to the respondent that the number should take into consideration all wards of the facility. If a range is given, probe further for an average number]***	1 Number of **referrals** in the **past** month ☐☐☐☐☐<br><br>**OR**<br><br>2 Number of **referrals** in the **past** year **(2012)** ☐☐☐☐☐<br><br>***[IF Q208 AND Q209 BOTH EQUAL 0, SKIP TO 211]***
210	When a PAC case comes in and you determine it's too severe for you to handle, what services do you give before you refer them on?<br><br>***[Interviewer: DO NOT PROMPT. Circle all that apply.]***	Stop the bleeding………………………. 1<br>Provide antibiotics………………………. 1<br>Provide pain killers…………………….. 1<br>Provide IV fluids for dehydration…….. 1<br>Provide counselling……………………… 1<br>We do not refer patients………….. 1<br>None of the above……………………… 1<br>Other (Specify) ___________________ 96
211	About how many post-abortion care patient referrals do you **RECEIVE** from other facilities in a typical month?	1 Number of **referrals received in typical** month ☐☐☐☐<br><br>**OR**<br><br>2 Number of **referrals received** in typical year ☐☐☐☐
212	***[Interviewer: Refer to the previous figures and fill in below before asking the respondent about the patient totals]***<br><br>So to confirm what you have just told me, in a **typical month** (or year), your facility treated<br><br>☐☐☐☐☐ Outpatients<br>☐☐☐☐☐ Inpatients<br><br>for abortion complications?<br><br>***[Interviewer: Please read out the total number of spontaneous and induced abortion patients seen at this facility in a typical month (Q204 and Q206)***<br><br>Is this number correct?<br>***[Interviewer: If correct, please insert again at right; if not then correct Q 204 and Q 206 and insert at right.]***	Summary per **month**<br><br>Outpatients……………. **1** ☐☐☐☐☐<br>Inpatients:………..……… **2** ☐☐☐☐☐<br>Total …………………….. **3** ☐☐☐☐☐<br><br>**OR**<br><br>Summary per **year**<br><br>Outpatients……………. **1** ☐☐☐☐☐<br>Inpatients:………..……… **2** ☐☐☐☐☐<br>Total …………………….. **3** ☐☐☐☐☐

213	***[Interviewer: Refer to the previous figures and fill in below before asking the respondent about the patient totals].***<br><br>So to confirm what you have just told me, in the **past month** (or **during the year** 2012) your facility treated:<br><br>[ ][ ][ ][ ][ ] Outpatients<br>[ ][ ][ ][ ][ ] Inpatients<br><br>for post-abortions complications?<br><br>***[Interviewer: Read out the total number of spontaneous and induced abortion patients seen at this facility in the past month (Q 205 and Q 207)].***<br><br>Is this number correct?<br>***[Interviewer: If correct, please insert again at right; if not then correct Q 205 and Q 207 and insert at right.]***	Summary in the **past month**<br>Outpatients……………. **1** [ ][ ][ ][ ][ ]<br>Inpatients:………..……… **2** [ ][ ][ ][ ][ ]<br>Total …………………….. **3** [ ][ ][ ][ ][ ]<br><br>**OR**<br><br>Summary in the **past year (2012)**<br><br>Outpatients……………. **1** [ ][ ][ ][ ][ ]<br>Inpatients:………..……… **2** [ ][ ][ ][ ][ ]<br>Total …………………….. **3** [ ][ ][ ][ ][ ]
214	What are the various procedures and measures used to treat abortion-complication patients at this facility?<br><br>***[Interviewer: Circle all applicable responses. DO NOT PROMPT respondent but probe to make sure you've captured all procedure types.] Ask is there any other procedure?***	D & E (dilation and evacuation)…………………… 1<br>D & C (dilation and curettage)……………………… 1<br>MVA (manual vacuum aspiration)………………… 1<br>EVA (electric vacuum aspiration)………………… 1<br>Medical abortion (e.g. Cytotec/misoprostol)……… 1<br>None…………………………………………… 1<br>Other (*specify*) ____________________ 96
215	Of these procedures, which is the **most commonly used** for treating patients with abortion complication at this facility?<br><br>***[Interviewer: Circle only one answer]***	D & E (dilation and evacuation)…………………… 1<br>D & C (dilation and curettage)……………………… 2<br>MVA (manual vacuum aspiration)………………… 3<br>EVA (electric vacuum aspiration)………………… 4<br>Medical abortion (e.g. Cytotec/misoprostol)……… 5<br>Other (specify) ____________________ 96<br>Not applicable………………………………… 99
216	In a typical month or year, what percentage of the post-abortion patients at this facility you think had these complications?<br><br>**[Interviewer: The percentages must add up to at least 100%. They may add up to more than 100% since a patient may suffer from more than one complication.]**	(see table below)

		%
1	Shock	
2	Sepsis	
3	Uterine perforation	
4	Hemorrhage	
5	Incomplete abortion	
6	Cervical/vaginal lacerations	
7	Bladder injury	
8	Intestinal injury	
96	Other (specify):	

Now I would like to ask you about the **major** characteristics of women seeking treatment for abortion complications, at this facility.

217	For every 100 women who are seen here for an abortion complication, about how many belong to each of the following age groups?<br>***[Interviewer: Read out the age groups. Make sure the total adds up to 100%.]***	(see table below)

	All Abortions
14 years or younger	
15-19 years	
20-29 years	
30-39 years	
40+ years	
Don't Know	
	100%

218	What marital status, you think is ***most commonly*** encountered?<br><br>***[Interviewer: Prompt if necessary]***		All Abortions	
		Married	1	
		Cohabitating	2	
		Single	3	
		Divorced/separated	4	
		Widowed	5	
		Don't Know	98	
219	What level of education, you think is ***most commonly*** encountered?<br><br>***[Interviewer: Prompt if necessary.***		All Abortions	
		No education	1	
		Primary	2	
		Secondary	3	
		Post-secondary	4	
		Don't Know	98	
220	How many children do you think these women ***most commonly*** already have?<br><br>***[Interviewer: Prompt if necessary. Circle only one.]***		All Abortions	
		Nulliparous	1	
		1-2	2	
		3-4	3	
		5 or more	4	
		Don't Know	98	
221	Would you say that these women ***more commonly*** live in urban or rural areas?<br><br>***[Interviewer: Circle only one.]***		All Abortions	
		Urban	1	
		Rural	2	
		Don't Know	98	
222	In this health facility, do you maintain information on the type of abortion complications treated?	Yes…………. 1<br>No………….. 2		

Module III: Postabortion Counseling

301	Does this facility offer postabortion contraceptive counseling to all, some or none of PAC patients? ***[Interviewer: Read out the alternatives]***	To all PAC patients……………. 1 → **[Skip to 303]**<br>To some of PAC patients……… 2<br>None……………………………. 3
302	For those patients to whom this facility doesn't offer post-abortion contraceptive counseling, what are the reasons?	____________<br>____________<br>____________<br>____________
303	Do you think women treated for abortion complications **should be given contraceptive counseling while still in the health facility?**	Yes…………………… 1 → **[Skip to 305]**<br>No……………………. 2<br>Sometimes…………. 3 → **[Skip to 305]**<br>Don't know/No opinion 98 → **[Skip to 305]**
304	Why do you think women treated for abortion complications should NOT receive family planning/contraceptive counseling?	____________<br>____________<br>____________<br>____________<br>____________
305	What topics **do you generally** cover in contraceptive counseling for post-abortion care patients?<br><br>***[Interviewer: DO NOT PROMPT. Multiple responses allowed.]***	Instructions on correct use of all methods………………… 1<br>Instructions on available methods………………………. 1<br>Instructions on traditional/natural methods only…………. 1<br>Instructions on side effects………………………………… 1<br>Advantages & disadvantages of each…………………….. 1<br>What to do in cases of method failure or forgetting pills…. 1<br>Abstinence…………………………………………….. 1<br>Other information you think should be included…………. 96<br>(specify) ____________<br>Not applicable/No counseling provided …………………… 99
306	What topics should be covered in the counseling on contraception for postabortion patients?<br><br>***[Interviewer: DO NOT PROMPT. Multiple responses allowed.]***	Instructions on correct use of all methods………………… 1<br>Instructions on available methods………………………. 1<br>Instructions on traditional/natural methods only…………. 1<br>Instructions on side effects………………………………… 1<br>Advantages & disadvantages of each…………………….. 1<br>What to do in case of method failure or forgetting pills…… 1<br>Abstinence……………………………………………. 1<br>Other information you think should be included…………. 96<br>(specify) ____________<br>No topics should be covered/No counseling 1
307	Do you involve men (OR PARTNERS?) in contraceptive selection	Yes……………… **1**<br>No……………….. **2**
308	IF YES, explain how you involve men , IF NO, explain why you do not involve them in contraceptive selection?	____________<br>____________<br>____________<br>____________<br>____________<br>____________<br>____________

309	Do you offer contraceptive methods on the premises of this facility?	Yes………………. **1**<br>No………………. **2** → **[Skip to Module 4]**
310	Which methods does your facility **commonly** offer to postabortion care patients?<br><br>***[Interviewer: DO NOT PROMPT. Multiple responses allowed.]***	Pills………………………………………………. 1<br>Injectables…………………………………………. 1<br>Implants……………………………………………. 1<br>Female sterilization………………………………. 1<br>IUD…………………………………………………. 1<br>Rhythm method (e.g. Moon beads) …………. 1<br>Condoms……………………………………………. 1<br>Vasectomy…………………………………………. 1<br>Emergency contraception………………………. 1<br>Withdrawal………………………………………. 1<br>Other (specify)_______________ 96<br>_______________<br>_______________
**[If provider selected all contraceptive methods in Q. 310, SKIP TO MODULE 4]**		
311	Why do you offer these methods and not others?<br><br>***[Interviewer: DO NOT PROMPT. Multiple responses allowed.]***<br>`	Those are the ones we have in stock………………………. 1<br>Provider preferences based on training……………………. 1<br>Provider preferences based on experience………………. 1<br>Provider preferences based on woman's charcteristics…. 1<br>Client preferences……………………………………………. 1<br>Not familiar enough with other methods……………………. 1<br>Religious reasons……………………………………………. 1<br>Funder's restriction…………………………………………. 1<br>Other (specify): _______________ 96

Module IV: General

Now we would like to ask you some general questions about your opinions about the Ugandan abortion law; the barriers on the provision of PAC, as well as how services can be improved.

401 Compared to your other services, about how much of a burden are postabortion care services on the care that your facility provides?

[Interviewer: READ THE LIST, check ONE response.]

A big burden	1	
A moderate burden	2	
A small burden	3	
Not a burden	4	→ **[Skip to Q403]**
Don't know/No opinion	98	→ **[Skip to Q403]**

402 Please tell me whether the following aspects of postabortion care are a burden to the service …

[Interviewer: READ THE LIST. Multiple responses allowed. Probe for any other responses.]

	Yes	No
a. The number of patients	1	2
b. The drain on equipment/ resources	1	2
c. Human resources/qualified personnel	1	2
d. Clients' inability to pay	1	2
e. Other (specify):	1	2

403 In your opinion, how could treatment for abortion complications be improved at this facility?

[Interviewer: Do not read the list. Multiple responses are allowed.]

a. Have a private room for postabortion care patients	1
b. Improve/expand infrastructure	1
c. Have more trained people available	1
d. Have available more commodities (MVA equipment, antibiotics, blood, pain medication)	1
e. Provide information on contraception and give methods	1
f. Provide protocols for caring for postabortion patients	1
g. Nothing is needed, current treatment is adequate	1
h. Other (specify):	96

404 Does the current Ugandan law permit abortion only to protect the life and health of the woman?

Yes…………………………….…………	1	
No………………………………..	2	→ **[Skip to Q406]**
Don't know /No opinion…………	3	→ **[Skip to Q407]**

405 Do you think the abortion law in Uganda should be changed?

Yes…………………………….…………	**1**	
No………………………………..	**2**	→ **[Skip to Q407]**
Don't know /No opinion…………	**98**	→ **[Skip to Q407]**

406 Under what circumstances should abortion be provided?

[Interviewer: DO NOT READ the list and circle all that apply. Multiple responses are allowed.]

Abortion should be provided:

a. On demand (if the woman requests for it)……………………	1
b. If the woman's health is at risk…………………………………	1
c. If the girl is still in school……………………………………….	1
d. Unmarried girl/woman …………….……………..……………	1
e. If the girl/woman cannot care for the child……………………	1
f. If the mother is HIV+…………………………………………….	1
g. Pregnancies after incest……………………………………….	1
h. Pregnancies after rape…………………………………………	1
i. Contraceptive failure………………………………………..	1
j. Abortion should not ever be provided………………………	1
k. Others (Specify)	96

[Interviewer: See Q.222. If respondent answered "YES," ask Q.407. Otherwise, go to END.]

407	You mentioned this facility maintains information about the type of abortion complications treated in this facility. Would it be possible to have access to that information or data?	Yes [1]<br>No [2] → **[END]**
408	***[Interviewer: Record the number of women treated in <u>2012 or last year available</u> according to the following abortion complications.***<br><br>***Obtain a photocopy or actual records, if available.***<br><br>***If a breakdown of complications is not available, just take the total number of PAC cases treated and thank the respondent/health records administrator.]***	see table below

	Number	Year of Data
Shock		
Sepsis		
Uterine perforation		
Hemorrhage		
Incomplete abortion		
Cervical/vaginal lacerations		
Bladder injury		
Intestinal injury		
Other (specify):		

Total number of PAC cases:	

END: Thank you for your cooperation
