# Supplementary material for: Incidence of Induced Abortion in Uganda, 2013: New Estimates Since 2003
Source: PLoS One. 2016 Nov 1;11(11):e0165812. doi: 10.1371/journal.pone.0165812 (PMC5089684; doi:10.1371/journal.pone.0165812)
Supplement: S2 Appendix — (PDF) [file pone.0165812.s002.pdf]

Investigation on Abortion and Postabortion Care (PAC)
Survey of Health Professionals in Uganda, 2013

My name is …….. and I am working with Makerere University/School of Public Health. The School in conjuction with the Guttmacher Institute, a USA research organization, is conducting a research project designed to update the conditions and the health implications of induced abortion, and to know how these have changed since 2003 when the first study on the incidence of abortion was carried out in Uganda. We would like to obtain your professional assessment of certain aspects of this practice.

We would greatly appreciate if you base your answers on your experience and knowledge, or if you lack actual experience, on your perception of the situation. Questions are asked separately about abortion practices in urban and rural areas, and separately about poor women and women who are relatively well-off (non-poor). If your experience or perceptions are insufficient to enable you to answer questions on a sub-group of women, please feel free to point this out. We urge you, however, to answer as fully as possible because your perceptions and opinions are valuable information where factual data are lacking. This interview will take at least 1 hour of your time.

Your responses to this questionnaire will be completely confidential and will be used for research purposes only. No personal reference will be made to your participation in this survey of health care professionals. We will combine your responses with those of other health professionals to describe the general picture of induced abortion practice and its health implications in Uganda. Participating in this survey poses little if any risk. Although we expect that the results of this study will help improve the nation's health care system, there will be no direct financial benefit from it to you personally.

Do I have your permission to proceed with the interview?

AGREE………………………………….☐

DO NOT AGREE…………………….….☐

Thank you

Study on Abortion and Postabortion Care (PAC)

Survey of Health Professionals in Uganda, 2013

001.	**Respondent Identification Number:**	☐☐☐
002.	**District:**	______________________ ☐☐
003.	**Region:**	______________________ ☐
004.	**Institution where respondent works:**	______________________
005.	**Date of Interview:**	☐☐ Day ☐☐ Month ☐☐☐☐ Year
006.	**Time Started:**	☐☐ hr ☐☐ min
007.	**Time Ended:**	☐☐ hr ☐☐ min
008.	**Interviewer's Name:**	______________________

NOTE: TO BE ADMINISTERED BY INTERVIEWER ONLY.

009.	**Result Code:**<br>***If not complete, please explain why:***<br>***[If facility is demolished or closed down, please try to find out when this occurred.]***	1 Complete<br>2 Incomplete<br>3 Refusal<br>4 Not found
	**Supervisor Signature:**	______________________

Interviewer's impression of the respondent's knowledge of rural areas ______________________

____

____

____

Module 1: Basic Information		
**Q #**	**Questions and Filters**	**Responses and Codes**
101	Sex of respondent	1 Male<br>2 Female
102	How old are you?	__ __ Years
103	Of the following categories, which describes your primary profession/current work?<br><br>***[Interviewer: READ LIST. If more than one applies, circle the category that accounts for the greatest proportion of the respondent's time.]***	1 Researcher<br>2 OBGYN specialist<br>3 Nurse<br>4 Midwife<br>5 Clinical Officer<br>6 Medical Officer/GP<br>7 Health Administrative Officer<br>8 Lawyer<br>9 Counselor/Social Work<br>10 Activist/Advocacy (s*pecify*) ____________<br>96 Other (*specify*) ____________
104	How long have you been working in this field?<br>***[Interviewer: Please record completed years. If less than one year, write "0".]***	__ __ Years
105	In which of the following sectors do you work primarily?<br>***[Interviewer: READ LIST. If the respondent works in more than one sector, circle the category corresponding to the sector where he/she contributes the most time. If the respondent works equally in both the private and the public sector, they can fill that in under "Other".]***	1 Public sector (government)<br>2 Private for profit<br>3 Private for non-profit (NGO)<br>96 Other *(specify)* ____________
106	In which area do you **currently** work--a rural or urban area?<br><br>***[Interviewer: If respondent works in both areas, circle the category of the area where he/she spends the most time. If the respondent works exactly part-time in both areas, then circle "Both"]***	1 Rural<br>2 Urban<br>3 Both
107	During the last five years, have you ever worked in a rural area for six months or more?	1 Yes<br>2 No → ***SKIP TO Q109***
108	In total, how long have you worked in rural areas?<br><br>***[Interviewer: Please record completed years. If less than one year, write "0".]***	__ __ Years

109	Have you been exposed to information on the practice of abortion…..<br><br>***[Interviewer: READ THE LIST. Multiple responses allowed. Please circle all that apply.]***	1	Through work in a public health facility
		1	Through work in a private health facility
		1	Through a non-medical framework (i.e. research, policy-making, counseling, advocacy, law, etc.)
		1	Through colleagues in any of the above settings
		96	Other *(specify)* : ______________

Module II: Provision of Induced Abortion

In Uganda, very little is known about the provision of induced abortion—that is, steps taken or procedures done to end a pregnancy. Induced abortion may be done at any gestation, and it may be done by both trained and untrained providers in safe and unsafe conditions. We would like to have your input about several aspects of this area of reproductive behavior, insofar as you are able to give an informed opinion.

[Interviewer: For this module, remind the respondent that all questions are to be answered with respect to the entire country. If the respondent indicates that he/she is unable to estimate on that level, then encourage the respondent to respond at a regional level, then denote in the margins that respondent has answered the question at a regional level. This should only be done if the respondent is completely unable to provide a country level response

201

202

There are various methods currently used in Uganda to try to induce abortion. From the list of methods I will read, please tell me which methods you know that are used **within this country,** regardless of the type of practitioner who may use it. I will first ask you about methods used in **urban areas** and then I will repeat the list for **rural areas**. Please let me know if there are any other methods you would like to specify.

[Interviewer: Please READ out each type of method for urban areas, circle the appropriate response for all that apply and then repeat the same list of methods for rural areas.]

DK=Do not know

Type of Method	Q.201 Urban Areas			Q.202 Rural Areas		
	Yes	No	DK	Yes	No	DK
**Standard methods**						
a. Manual vacuum aspiration (MVA)	1	2	98	1	2	98
b. Dilation and curettage (D&C)	1	2	98	1	2	98
c. Dilation and Evacuation (D&E)	1	2	98	1	2	98
**Oral introduction of drugs, solutions or other substances (i.e. through the mouth)**						
d. Misoprostol (e.g. Cytotec)	1	2	98	1	2	98
e. Other hormonal drugs (i.e. contraceptive pills)	1	2	98	1	2	98
f. Herbs /Teas /Solutions	1	2	98	1	2	98
**Vaginal introduction of drugs, solutions or other materials**						
g. Misoprostol (e.g. Cytotec)	1	2	98	1	2	98
h. Other hormonal drugs (i.e. contraceptive pills)	1	2	98	1	2	98
i. Herbs/Teas/ Solutions (Using form of insertion)	1	2	98	1	2	98
j. Catheter	1	2	98	1	2	98
k. Caustic agents (e.g. Washing detergent)	1	2	98	1	2	98
l. Overdose of pharmaceuticals (e.g. Quinine, Fansider)	1	2	98	1	2	98
m. Other solid objects (*urban*)_________________________	1	2	98	1	2	98
Other solid objects (*rural*) _________________________						
**Physical methods**						
n. Heavy massage or pressure on abdomen, physical blows, falling	1	2	98	1	2	98
o. Excessive exercise	1	2	98	1	2	98
**Other methods**						
p. Injectables specify_______________________________	1	2	98	1	2	98
q. Other means (Specify) any additional method/s not listed above)	1	2	98	1	2	98
*(urban)*____						
(rural)						

203

204

What do you think is the MOST common method used by:

	Q203. Urban Areas	Q204. Rural Areas
TBA/Traditional healer		
Clinical officer/Nurse/Midwife		
Doctor		
Woman herself (self-induction)		

[Interviewer:use the letters in Q.202 or enter "98" if the person cannot provide a response]

The following questions are asked separately about women who live in urban and rural areas. Each one asks you to consider two broad socioeconomic groups – the poor and the relatively well-off (non-poor). ***Considering this country as a whole, I want you to give us your opinion on the following.***

[Interviewer*: There are no exact definitions for "poor" and "non-poor," but by "poor" we mean women with seven or less years of schooling.* ***Only if necessary****, explain that poor women are those who have economic difficulties to afford the costs of a safe abortion.*

URBAN AREAS

205 206 **[Interviewer: Please read out each type of provider for urban poor then reread list for urban non-poor. Probe for an answer if "don't know".]**

C=Commonly, S=Sometimes, R=Rarely, N=Never, DK=Don't know

Q.205 Considering first **URBAN** areas, indicate whether, in your opinion, each of the following types of provider is commonly, sometimes, rarely or never used by **poor** women seeking an abortion.

Q.206 And now for **non-poor URBAN** women. Please indicate whether each of the following types of provider is commonly, sometimes, rarely or never used by non poor women seeking an abortion.

Type of provider	Urban poor women							Urban non-poor women						
		C	S	R/N		DK			C	S	R/N		DK	
a. Doctor		1	2	3		98			1	2	3		98	
b. Clinical officer		1	2	3		98			1	2	3		98	
c. Nurse		1	2	3		98			1	2	3		98	
d.Certified/Trained Midwife		1	2	3		98			1	2	3		98	
e. Pharmacist/Dispenser/drugstore/chemist		1	2	3		98			1	2	3		98	
f. TBA/traditional healer		1	2	3		98			1	2	3		98	
g. Woman - self-induced		1	2	3		98			1	2	3		98	
h. Other *(poor)* : ______________		1	2	3		98			1	2	3		98	
Other *(non-poor)* :														

207 208 ***[Interviewer: Please check with <u>Q 205 and Q206 and request an amount or price range for only those providers who were rated as used commonly or used sometimes.</u> Probe if the respondent answers "don't know."]***

Q.207. How much do you think **poor women in URBAN areas** pay for a first-trimester abortion (14 weeks or less) from each of the following providers.

Q.208. Now, let's turn to **non-poor women**. How much do you think they pay for first-trimester abortions from each of these providers?

Type of Provider	Urban poor women	Urban non-poor women
a. Doctor		
b. Clinical officer		
c. Nurse		
d.Certified/Trained Midwife		
e. Pharmacist/Dispenser/drugstore/chemist		
f. TBA/traditional healer		
g. Woman - self-induced		
h. Other *(poor)* : ______________________________		
Other *(non-poor)* :		

209-210	**[Interviewer: Please read out each type of provider. Confirm that all providers sum to 100%. If they do not, probe for a correction, and adjust the answers below.]**	**Q.209** What percent of all induced abortions do you think are being performed by the following providers for **poor URBAN** women? Please note that all percentages must add to 100%	**Q.210** Now, let's turn to non-poor women. What percent of all induced abortions do you think are being performed by the following providers for **non- poor URBAN** women?
	**Type of Provider**	**Urban poor women**	**Urban non-poor women**
	a. Doctor	. %	%
	b. Clinical officer	%	%
	c. Nurse	%	%
	d.Certified/Trained Midwife	%	%
	e. Pharmacist/Dispenser/drugstore/chemist	%	%
	f. TBA/traditional healer	%	%
	g. Woman - self-induced	%	%
	h. Other *(poor)* :<br>Other *(non-poor)* :	%	%
	TOTAL	100%	100%

The next set of questions focuses on women living in ***rural*** *areas in Uganda.*

RURAL AREAS

211<br>212	**[Interviewer: Please read out each type of provider for rural poor then reread list for rural non-poor.<br>Probe for an answer if "don't know".]**<br>C=Commonly, S=Sometimes, R=Rarely, N=Never, DK=Don't know	**Q.211** Please indicate if each of the following types of provider is commonly, sometimes, rarely or never used by poor rural women seeking an abortion.						**Q.212** Now considering **non-poor RURAL women**. Please indicate whether each of the following type of provider is commonly, sometimes, rarely or never used by non poor women seeking an abortion.					
	**Type of provider**	**Rural poor women**						**Rural non-poor women**					
			C	S	R/N		DK		C	S	R/N		DK
	a. Doctor		1	2	3		98		1	2	3		98
	b. Clinical officer		1	2	3		98		1	2	3		98
	c. Nurse		1	2	3		98		1	2	3		98
	d.Certified/Trained Midwife		1	2	3		98		1	2	3		98
	e. Pharmacist/Dispenser/drugstore/chemist		1	2	3		98		1	2	3		98
	f. TBA/traditional healer		1	2	3		98		1	2	3		98
	g. Woman - self-induced		1	2	3		98		1	2	3		98
	h. Other *(poor)* : ___________________<br>Other *(non-poor)* :		1	2	3		98		1	2	3		98

213<br>214	***[Interviewer: Please check with <u>Q 211 and Q212 and request an amount or price range for only those providers who were rated as used commonly or used sometimes</u><br>Probe for an answer if the respondent answers "don't know."]***	**Q.213.** How much do you think **poor women in RURAL areas** pay for a first trimester abortion (14 weeks of less) from each of the following providers.	**Q.214**. Now, focus on **non-poor** women. How much do you think they pay for first trimester abortions from each of these providers?
	**Type of Provider**	**Rural poor women**	**Rural non-poor women**
	a. Doctor		
	b. Clinical officer		
	c. Nurse		
	d.Certified/Trained Midwife		
	e. Pharmacist/Dispenser/drugstore/chemist		
	f. TBA/traditional healer		
	g. Woman - self-induced		
	h. Other *(poor)* : ________________________________<br>Other *(non-poor)* :		

215-216	**[Interviewer: Please read out each type of provider. Confirm that all providers sum to 100%. If they do not, probe for a correction, and adjust the answers below.]**	**Q.215** What percent of all induced abortions do you think are being performed by the following providers for **poor RURAL** women? Please note that all percentages must add to 100%	**Q.216** Now, let's turn to non-poor women. What percent of all induced abortions do you think are being performed by the following providers for **non- poor RURAL** women?
	**Type of Provider**	**Rural poor women**	**Rural non-poor women**
	a. Doctor	. %	%
	b. Clinical officer	%	%
	c. Nurse	%	%
	d.Certified/Trained Midwife	%	%
	e. Pharmacist/Dispenser/drugstore/chemist	%	%
	f. TBA/traditional healer	%	%
	g. Woman - self-induced	%	%
	h. Other *(poor)* :<br>Other *(non-poor)* :	%	%
	TOTAL	100%	100%

Module III: Abortion Complications

*Now, I would like to talk to you about abortion complications. As you know, complications from an induced abortion range in severity. When we speak of abortion complications, we are referring to those consequences **severe enough to need treatment in a health facility,** regardless of whether the woman obtains health care. We define complications to include not only extremely serious cases such as sepsis or a perforated uterus, but also those cases termed "incomplete abortions." Incomplete abortions are usually identified by heavy bleeding and present a somewhat less severe health risk to the woman, but still require hospitalization. In answering the following questions, please keep this definition in mind.*

301 Considering **urban areas**, please indicate which forms of short term health complications apart from death, result commonly, sometimes or never from induced abortion in Uganda.

Now considering **rural areas in Uganda**....

[Interviewer: Please read each type of complication and circle the respondent's answers for each type of complication]

C=commonly; S=sometimes; N=never	Urban areas			Rural Areas		
	C	S	N	C	S	N
**a**. Incomplete abortion	1	2	3	1	2	3
**b**. Excessive loss of blood	1	2	3	1	2	3
**c**. Infection of the uterus and/or surrounding areas	1	2	3	1	2	3
**d**. Sepsis or septic shock	1	2	3	1	2	3
**e**. Damage to gut (intestines)	1	2	3	1	2	3
**f**. Damage to vagina and cervix	1	2	3	1	2	3
**g**. Damage to uterus (e.g. Perforation)	1	2	3	1	2	3
**h**. Pain in lower abdomen	1	2	3	1	2	3
**i**. Other	1	2	3	1	2	3

(specify, urban) ______________________________

(specify rural) ______________________________

We are now asking a set of questions about women living in urban areas in Uganda.

URBAN AREAS

302-303 **[Interviewer: Ask for each type of provider separately; insert a number for each type of provider, even though it might be '0'.]**

Q.302 Think about **poor** women in **URBAN** areas. Out of 10 **poor urban** women who have an abortion performed by each type of provider that I will mention, how many would experience a medical complication that should receive medical treatment in a health facility?

Q.303 Now, consider **non-poor URBAN** women. Out of 10 **non-poor urban** who have an abortion performed by the following providers, how many would experience and medical complication that should receive medical treatment in a health facility?

Provider	Out of 10 poor urban women	Out of 10 non-poor urban women
a. Doctor		
b. Clinical officer		
c. Nurse		
d.Certified/Trained Midwife		
e. Pharmacist/ Dispenser /drugstore/chemist		
f. TBA/traditional healer		
g. Woman - self-induced		
h. Other *(poor)* : ____________		
Other *(non-poor)* :		

304 a. Think about **poor** women in **URBAN** areas: out of 10 poor urban women who experience a **medical complication** due to an induced abortion, how many do you think **actually get treated** by a trained person in a health facility?

[ ][ ] **Urban poor** women out of 10 treated in a health facility

b. What would the number be for **non-poor** women living in an **URBAN** area?

[ ][ ] **Urban non-poor** women out of 10 treated in a health facility

305-306	**[Interviewer: Read out each type of facility for both urban poor and urban non-poor. Probe for an answer if "don't know", only accept if necessary.]**<br>C=commonly R=rarely<br>S=sometimes N=never<br>DK=don't know	**Q.305** How often do **poor URBAN** women go to the following places to be treated for medical complications that result from induced abortion: commonly, sometimes, rarely/never?	**Q.306** Now thinking about **non-poor URBAN** women. How often do they go to the these places to be treated for medical complications that result from induced abortion: commonly, sometimes, rarely/never?

**Type of provider/facility**	C	S	R/N	DK	C	S	R/N	DK
a. Doctor	1	2	3	98	1	2	3	98
b. Clinical officer	1	2	3	98	1	2	3	98
c. Nurse	1	2	3	98	1	2	3	98
d.Certified/Trained Midwife	1	2	3	98	1	2	3	98
e. Pharmacist/Dispenser/drugstore/chemist	1	2	3	98	1	2	3	98
f. TBA/traditional healer	1	2	3	98	1	2	3	98
g. Woman - self-treated	1	2	3	98	1	2	3	98
h. Other *(poor)* : ________________<br>Other *(non-poor)* :	1	2	3	98	1	2	3	98

We are now asking a set of questions about women living in rural areas.

RURAL AREAS

307<br>308	**[Interviewer: Ask for each type of provider separately; insert a number in each column, even though it might be '0'.]**	**Q.307** Think about **poor** women in **RURAL** areas. Out of 10 **poor RURAL** women who have an abortion performed by each type of provider that I will mention, how many would experience a medical complication that should receive medical treatment in a health facility?	**Q.308** Now, consider **non-poor RURAL** women. Out of 10 **non-poor RURAL** who have an abortion performed by the following providers, how many would experience and medical complication that should receive medical treatment in a health facility?

	**Out of 10 poor rural women**	**Out of 10 non-poor rural women**
a. Doctor		
b. Clinical officer		
c. Nurse		
d.Certified/Trained Midwife		
e. Pharmacist/ Dispenser /drugstore/chemist		
f. TBA/traditional healer		
g. Woman - self-induced		
h. Other *(poor)* : ________________<br>Other *(non-poor)* :		

309	a. Think about **poor** women in **RURAL** areas: out of 10 poor rural women who experience a **medical complication** due to an induced abortion, how many do you think **actually get treated** by a trained person in a health facility?		**Rural poor** women out of 10 treated in a health facility
	b. What would the number be for **non-poor** women living in a **RURAL** area?		**Rural non-poor** women out of 10 treated in a health facility

310<br>311	**[Interviewer: Read out each type of facility for both rural poor and rural non-poor. Probe for an answer if "don't know", only accept if necessary.]**<br><br>C=commonly R=rarely<br>S=sometimes N=never	**Q.310** How often do **poor RURAL** women go to the following places to be treated for medical complications that result from induced abortion: commonly, sometimes, rarely/never?	**Q.311** Now thinking about **non-poor RURAL** women. How often do they go to the these places to be treated for medical complications that result from induced abortion: commonly, sometimes, rarely/never?

Type of provider/facility	C	S	R/N	DK	C	S	R/N	DK
a. Doctor	1	2	3	98	1	2	3	98
b. Clinical officer	1	2	3	98	1	2	3	98
c. Nurse	1	2	3	98	1	2	3	98
d.Certified/Trained Midwife	1	2	3	98	1	2	3	98
e. Pharmacist/Dispenser/drugstore/chemist	1	2	3	98	1	2	3	98
f. TBA/traditional healer	1	2	3	98	1	2	3	98
g. Woman - self-treated	1	2	3	98	1	2	3	98
h. Other *(poor)* : ____________<br>Other *(non-poor)* :	1	2	3	98	1	2	3	98

Now I will ask you just a question about live births attended by a trained provider at a facility.

		% Live births attended in HF	
		1. Urban Women	2. Rural Women
312	Of all urban women who deliver a live born child in this country, what percent would you say go to a public or private facility that is staffed with a trained provider to assist the delivery?<br><br>**And what would the percentage be for rural women in the whole country?**	_______	_______

313 For the following, please indicate whether it is never/rarely, sometimes, or always a barrier to the use of post abortion care services.

[Interviewer: READ THE LIST and indicate in each case (A) Always, (S) Sometimes, (R) Rarely, or (N) Never

	Barriers for use of PAC services	A	S	R/N	DK
a.	Cost to woman	1	2	3	98
b.	Distance/ transportation	1	2	3	98
c.	Inadequate training of providers	1	2	3	98
d.	Inadequate space/equipment at facility	1	2	3	98
e.	Under-staffing at facility	1	2	3	98
f.	Hostile/unfriendly provider attitudes	1	2	3	98
g.	Lack of patient information on services	1	2	3	98
h.	Husband/family objections	1	2	3	98
i.	Fear/stigma	1	2	3	98
j.	Delay in identifying danger signs	1	2	3	98
k.	Other (specify):	1	2	3	98

Module IV: Use of misoprostol

Misoprostol (or Cytotec) is increasingly becoming available in Uganda and there is the possibility that it is also being used for a number of purposes. I would like to ask your views about use of misoprostol for induced abortion in Uganda.

401	As far as you are aware, is misoprostol currently being used in Uganda to induce abortion?	1<br>2<br>98	Yes<br>No → ***SKIP TO SECTION 5***<br>Don't know → ***SKIP TO SECTION 5***
402	For how long has misoprostol been used in Uganda to induce abortion?<br>**[Interviewer: If less than one year, write "0"]**	__ __<br>98	years ago<br>Don't know
403	As far as you are aware, where do you think misoprostol is most commonly used: in urban areas, rural areas or equally both?	1<br>2<br>3<br>98	More in urban areas<br>More in rural areas<br>Equally in both areas<br>Don't know
404	As far as you are aware, where do you think misoprostol is provided or sold?<br>**[Interviewer: Do not read list. Multiple responses allowed. Please circle all that apply.]**	1<br>1<br>1<br>1<br>1<br>1<br>1<br>96<br>98	Doctors' private practice<br>Clinical Officers' private practice<br>Nurses'/midwives' private practice<br>Traditional birth attendants/Herbalists<br>Pharmacies<br>Chemist/Drug vending stores<br>Hospitals/Health facility<br>Other (specify):____________________<br>Don´t know

405 Regardless of urban or rural area, how frequently do you think misoprostol is used or "provided" to terminate a pregnancy by the following people: commonly, sometimes, rarely or never?

[Interviewer: Read each person listed below and circle the the appropriate response]

**Type of Provider**	Commonly	Sometimes	Rarely/ Never	Don't Know
a. Medical doctor	1	2	3	98
b. Clinical Officer	1	2	3	98
c. Nurse/midwife	1	2	3	98
d. Traditional birth attendant	1	2	3	98
e. Pharmacist	1	2	3	98
f. Chemist/drug vendor	1	2	3	98
g. Woman (self-induced)	1	2	3	98
h. Partner/Spouse	1	2	3	98
i. Friends/family member	1	2	3	98
j. Other *(specify)* : __________	1	2	3	98

[Interviewer: Check Q405 and for each category of provider mentioned as commonly or sometimes use misoprostol, ask Q406.]

406 How much do you think a woman in Uganda pays for an abortion performed using misoprostol from each of the following sources?

**Type of Provider**	Amount in Shillings	Don't Know
a. Medical doctor		98
b. Clinical Officer		98
c. Nurse/midwife		98
d. Traditional birth attendant		98
e. Pharmacist		98
f. Chemist/drug vendor		98
g. Other *(specify)* : ________________________________		98

407 As you know, each medication should be administered in the adequate dose to be effective and safe. How often do you think the following people who provide misoprostol for the purposes of pregnancy termination, generally know the correct dose: commonly, sometimes, rarely or never?
[Interviewer: Read each provider/person and circle the appropriate response.]

**Type of Provider**	Commonly	Sometimes	Rarely/ Never	Don't Know
a. Medical doctor	1	2	3	98
b. Clinical Officer	1	2	3	98
c. Nurse/midwife	1	2	3	98
d. Traditional birth attendant	1	2	3	98
e. Pharmacist	1	2	3	98
f. Chemist/drug vendor	1	2	3	98
g. Woman (self-induced)	1	2	3	98
h. Partner/Spouse	1	2	3	98
i, Friends/family member	1	2	3	98
j. Other *(specify)* : ______________________	1	2	3	98

408	Out of ten **women** who have an abortion by the use of misoprostol, how many do you think would experience a medical complication that should receive medical treatment in a health facility?<br><br>**[Interviewer: Please refer to definition of complications in the beginning of Section 3]**	__ __	Women out of 10 experience complications
409	Out of 10 **women** who experience a medical complication from an abortion performed by the use of misoprostol, how many do you think actually get treated by a trained person in a health facility?	__ __	Women out of 10 treated in a health facility

Module V: Postabortion Counselling

501	As you know, some women become pregnant while they are using contraceptive methods.<br><br>In your opinion, which methods were women with unintended pregnancies using at the time of conception?<br><br>**[Interviewer: DO NOT PROMPT. Multiple responses are allowed.]**<br><br>**Probe for other methods.**<br>(Are there other methods? What else...)	1 Pills<br>1 Injectables<br>1 Implants<br>1 IUD<br>1 Male condom<br>1 Female condom<br>1 Diaphram<br>1 Tubal ligation (Female sterilization)<br>1 Vasectomy (Male sterilization)<br>1 Withdrawal<br>1 Rhythm method (e.g. Moon beads)<br>1 Spermicides<br>1 Emergency contraception<br>1 Abstinence<br>1 No method<br>1 Lactational amenorrhea/Breast feeding<br>96 Other (specify):____________________
502	Do you think providers of PAC services offer contraceptive counselling to all, some, or none of their patients?	1 All<br>2 Some<br>3 None<br>98 Don't know
503	Do you think that all abortion patients should be offered postabortion contraceptive counselling while they are still at the health facility?	1 Yes<br>2 No
504	Do you think that all abortion patients should be offered a contraceptive method while they are still at the health facility?	1 Yes<br>2 No<br>3 Sometimes
505	In your opinion, which methods are most appropriate for post abortion care patients?<br><br>**[Interviewer: DO NOT PROMPT. Multiple responses are allowed.]**<br><br>**Probe for other methods.**<br>(Are there other methods? What else...)	1 Pills<br>1 Injectables<br>1 Implants<br>1 IUD<br>1 Male condom<br>1 Female condom<br>1 Diaphram<br>1 Tubal ligation (Female sterilization)<br>1 Vasectomy (Male sterilization)<br>1 Withdrawal<br>1 Rhythm method (e.g. Moon beads)<br>1 Spermicides<br>1 Emergency contraception<br>1 Abstinence<br>1 No method<br>96 Other (specify):_____________________

Module VI: Opinions

601 Comparing the problem of medical complications due to unsafe abortion with other health problems of pregnant women in Uganda, would you say that unsafe abortion is:

[Interviewer: Read list and circle one response.]

1	A serious problem
2	A minor problem
3	Not a problem

602 I am going to read some of the most common reasons women in Uganda seek to have an induced abortion. Please rate each reason as a frequent, somewhat frequent or infrequent reason for abortion.

[Interviewer: Please read each reason and ask its frequency]

Reason	Frequent	Somewhat frequent	Infrequent	Don't know
a. The woman is unmarried	1	2	3	98
b. Economic reasons	1	2	3	98
c. The pregnancy is unplanned or unwanted	1	2	3	98
d. The pregnancy may affect the woman's health	1	2	3	98
e. The pregnancy threatens the woman's life	1	2	3	98
f. Indication that child will have mental or physical anomalies	1	2	3	98
g. Pregnancy resulted from incest	1	2	3	98
h. The pregnancy is the result of rape	1	2	3	98
i. Other *(specify):*	1	2	3	98

603 Please indicate if any of the recommendations I am going to list could be used in Uganda to reduce the number of unsafe abortions and to lower the level of abortion in general:

[Interviewer: Please read each recommendation.]

	Yes	No	Maybe	Don't know
a. Improve sex education in schools	1	2	3	98
b. Promote abstinence	1	2	3	98
c. Publicize the health risks involved in unsafe abortion	1	2	3	98
d. Provide contraceptive counselling post-delivery and postabortion	1	2	3	98
e. Increase availability of family planning services	1	2	3	98
f. Improve access to effective contraception for young and unmarried women	1	2	3	98

604 Are there any other recommendations that you would make to reduce the number of unsafe abortions in Uganda? What are they?

[Interviewer: Record response(s) in the space provided. If no other recommendations, write "None."]

605 Do you feel that the laws of Uganda on abortion should be modified?

1	Yes
2	No **[END]**
3	No opinion **[END]**

606	Under what circumstances do you think that abortion should be provided?<br><br>**[Interviewer: DO NOT READ the list and circle all that apply. Multiple responses are allowed.]**<br><br>**[Interviewer: Record other response(s) in the space provided.]**		**Abortion should be provided:**
		1	On demand (if the woman requests for it)
		1	If the woman's health is at risk
		1	If the girl is still in school
		1	Unmarried girl/woman
		1	If the girl/woman cannot care for the child
		1	If the mother is HIV+
		1	Pregnancies after incest
		1	Pregnancies after rape
		1	Contraceptive failure
		1	Abortion should not ever be provided
		96	Others (Specify) _______________________<br>___________________________

Thank you very much for your time. Your views are very important and will help make health services better for women. If you are interested in receiving the final publication based on this survey after it is completed, for women.we will be happy to provide it to you.

1 YES, agrees to be contacted to receive publication

2 NO, does not agree to be contacted

[Inteviewer: Please record contact information in a place separate from the questionnaire]

Interviewer observations: ____
____
____
____
____
____
____
